# Marine Fungi from the Sponge *Grantia compressa*: Biodiversity, Chemodiversity, and Biotechnological Potential

**DOI:** 10.3390/md17040220

**Published:** 2019-04-11

**Authors:** Elena Bovio, Laura Garzoli, Anna Poli, Anna Luganini, Pietro Villa, Rosario Musumeci, Grace P. McCormack, Clementina E. Cocuzza, Giorgio Gribaudo, Mohamed Mehiri, Giovanna C. Varese

**Affiliations:** 1 Mycotheca Universitatis Taurinensis, Department of Life Sciences and Systems Biology, University of Turin, Viale Mattioli 25, 10125 Turin, Italy; elena.bovio@unito.it (E.B.); laura.garzoli@unito.it (L.G.); anna.poli@unito.it (A.P.); 2 Laboratory of Microbiology and Virology, Department of Life Sciences and Systems Biology, University of Turin, Via Accademia Albertina 13, 10123 Turin, Italy; anna.luganini@unito.it (A.L.); giorgio.gribaudo@unito.it (G.G.); 3 Laboratory of Clinical Microbiology and Virology, Department of Medicine, University of Milano-Bicocca, via Cadore 48, 20900 Monza, Italy; p.villa18@campus.unimib.it (P.V.); rosario.musumeci@unimib.it (R.M.); clementina.cocuzza@unimib.it (C.E.C.); 4 Zoology, Ryan Institute, School of Natural Sciences, National University of Ireland Galway, University Road, Galway H91 TK33, Ireland; grace.mccormack@nuigalway.ie; 5 University Nice Côte d’Azur, CNRS, Nice Institute of Chemistry, UMR 7272, Marine Natural Products Team, 60103 Nice, France

**Keywords:** sponges, marine fungi, biodiversity, *Eurotium chevalieri*, OSMAC, bioactive compounds, antibacterial, antiviral

## Abstract

The emergence of antibiotic resistance and viruses with high epidemic potential made unexplored marine environments an appealing target source for new metabolites. Marine fungi represent one of the most suitable sources for the discovery of new compounds. Thus, the aim of this work was (i) to isolate and identify fungi associated with the Atlantic sponge *Grantia compressa*; (ii) to study the fungal metabolites by applying the OSMAC approach (one strain; many compounds); (iii) to test fungal compounds for their antimicrobial activities. Twenty-one fungal strains (17 taxa) were isolated from *G. compressa*. The OSMAC approach revealed an astonishing metabolic diversity in the marine fungus *Eurotium chevalieri* MUT 2316, from which 10 compounds were extracted, isolated, and characterized. All metabolites were tested against viruses and bacteria (reference and multidrug-resistant strains). Dihydroauroglaucin completely inhibited the replication of influenza A virus; as for herpes simplex virus 1, total inhibition of replication was observed for both physcion and neoechinulin D. Six out of 10 compounds were active against Gram-positive bacteria with isodihydroauroglaucin being the most promising compound (minimal inhibitory concentration (MIC) 4–64 µg/mL) with bactericidal activity. Overall, *G. compressa* proved to be an outstanding source of fungal diversity. Marine fungi were capable of producing different metabolites; in particular, the compounds isolated from *E. chevalieri* showed promising bioactivity against well-known and emerging pathogens.

## 1. Introduction

In the last few decades, antibiotic resistance significantly increased, while the discovery of new molecules suitable for the therapy of infectious diseases slowed down [1]; therefore, finding new antimicrobial compounds became a priority. Natural products of terrestrial origins are always considered the primary sources of bioactive molecules [2]. However, unexplored environments and organisms, such as those inhabiting the sea, became appealing for the discovery of new compounds. The number of new compounds of marine origin is remarkably high, e.g., in 2016 alone, 1277 new natural products with promising biomedical applications were described. In this regard, marine fungi were responsible for producing 36% of these newly described compounds [3]. With regard to ease of production of compounds of interest, fungi are attractive sources of natural products when compared to macroorganisms. As an example, large quantities (1000 kg, wet weight) of the tunicate *Ecteinascidia turbinata* were necessary to obtain 1 g of trabectedin, a promising anticancer compound [4]; today, its production is only feasible by bacterial fermentation to obtain a precursor of trabectedin [5]. On the contrary, many marine fungi can be easily cultivated to achieve a high yield of secondary metabolites, thus representing a sustainable alternative to chemical synthesis [4]. To expand the range of secondary metabolites produced by marine fungi, the OSMAC (one strain; many compounds) approach [6], based on the modulation of culture conditions, is widely recognized as an effective strategy to find new compounds [2,7,8,9,10,11]. Indeed, one way to find new metabolites is the simulation of the native environment of the marine fungi, including their interactions with other microorganisms [8,12,13]. However, the extent to which the OSMAC approach induces the stimulation of secondary metabolites is still the subject of discussion. Experimental data highlighted that both the modulation of culture conditions and the screening of different fungal species contribute to increasing the rate of discovery of new metabolites [11]. Thus, marine fungi represent a large reservoir of uncharacterized natural products to which the OSMAC approach is barely applied [8]. Furthermore, they are one of the lesser studied groups of microorganisms, with only 1206 obligate and facultative marine species characterized out of the 10,000 estimated [14,15]; however, their biodiversity is suggested as being much higher [16].

Marine sponges, sometimes referred to as microbial fermenters, are an outstanding source of highly diverse microbial communities [17], including new fungal species [18]. Several sponge-derived fungi produced compounds with different biological activities, including anticancer, anti-inflammatory, and anti-feeding properties for marine organisms [19].

The aim of this study was to (i) investigate for the first time the culturable fungal community associated with the Atlantic sponge *Grantia compressa* (Grantiidae, Leucosolenida); (ii) induce metabolite production by applying the OSMAC approach; (iii) characterize the promising marine sponge-associated fungus *Eurotium chevalieri* MUT 2316; and (iv) assess the antimicrobial potential of the isolated metabolites.

Overall, the OSMAC approach highlighted the fungal strains that produced different secondary metabolites and the media that were more suitable to increase chemical diversity. Among the marine fungi isolated, *E. chevalieri* MUT 2316 was further studied and yielded 10 compounds with promising antibacterial and antiviral activities, some of which are also present in *E. chevalieri* KUFA 0006 [20]. 

## 2. Materials and Methods

### 2.1. Sample Materials and Fungal Isolation

Three specimens of the Atlantic sponge *G. compressa* (identified by Prof G. P. McCormack, NUI, Ireland) were collected at Coranroo (West Coast of Ireland; WGS84 north (N) 53°09’100”, west (W) 009°00’550”) at a site with rapid water flow over limestone rocks. The samples were kept at 4 °C until laboratory analyses were performed. To remove contaminants and sediments, the samples were surface sterilized with ethanol 70% (30 s) and serially washed in artificial sterile sea water (SW; sea salts 30 g, Sigma-Aldrich, Saint Louis, USA, up to 1 L of deionized (DI) H_2_O). For fungal isolation, two different techniques were applied, as follows:

(1) Homogenization (HO): 5 g of each sponge sample was homogenized (homogenizer blade Sterilmixer II, PBI International) in SW (1:10 *w*/*v*). One milliliter of the suspension was plated onto Petri dishes (15 cm Ø) containing (i) Corn Meal Agar SW (CMASW; corn meal 2 g, agar 15 g, sea salts 30 g, Sigma-Aldrich, Saint Louis, USA, up to 1 L of DI H_2_O); (ii) Malt Extract Agar (MEA; malt 20 g, glucose 20 g, peptone 2 g, agar 15 g, Sigma-Aldrich, Saint Louis, USA, up to 1 L of DI H_2_O); or (iii) Gelatin Agar SW (GASW; gelatin 20 g, sea salts 30 g, agar 15 g, Sigma-Aldrich, Saint Louis, USA, up to 1 L of DI H_2_O) to mimic sponge composition. Plates were incubated in the dark at 15 °C and 25 °C, and checked daily for three weeks. Five replicates for each condition were performed.

(2) Direct plating (DP): sponges were cut into 20 pieces (about 0.5 cm^3^) and plated onto Petri dishes (6 cm Ø) containing CMASW or SW Agar (SWA; sea salts 30 g, agar 15 g, Sigma-Aldrich, Saint Louis, USA, up to 1 L of DI H_2_O). Plates were incubated in the dark at 15 °C and 25 °C, and checked daily for three weeks. Five replicates for each condition were performed.

All media were supplemented with an antibiotic mix (gentamicin sulfate 40 mg/L, piperacillin plus tazobactam 11 mg/L) to avoid bacterial growth.

### 2.2. Fungal Identification

Fungi were identified by both their morpho-physiological features [21,22,23] and molecular data. Briefly, fungal specimens were firstly identified to genus level based on their morphology in order to use the most appropriate primers for molecular analysis. Subsequently, the genomic DNA was extracted using a NucleoSpin kit (Macherey Nagel GmbH, Duren, DE, USA), according to the manufacturer’s instructions. The primers and PCR conditions used to amplify specific molecular markers are listed in Table S1 (Supplementary Materials). The taxonomic position of sterile mycelia (lacking any reproductive structures) presenting low homology with sequences available in public databases was inferred by phylogenetic analysis; for the genus *Emericellopsis*, datasets based on sequences from ribosomal internal transcribed spacer region (ITS) and partial beta-tubulin gene (TUB) were created (Table S2, Supplementary Materials). Multiple alignments of each gene region were generated using MUSCLE (default conditions for gap openings and gap extension penalties), implemented in MEGA v. 7.0 (Molecular Evolutionary Genetics Analysis), visually inspected, and manually refined to discard ambiguously aligned regions. Then, alignments were concatenated into a single data matrix with SequenceMatrix v. 1.8 [24]. The appropriate evolutionary model under the Akaike information criterion (AIC) was determined with jModelTest 2 [25]. Bayesian inference (BI) was performed with MrBayes 3.2.2 [26] under a General Time Reversible with a proportion of Invariable sites plus discrete Gamma evolutionary model (GTR + I + G; best model). The alignment was run for 10 million generations with two independent runs, each containing four Markov chain Monte Carlo (MCMC) models and sampling every 1000 iterations. The first 2500 trees were discarded as “burn-in” (25%). Using the Sumt function of MrBayes, a consensus tree was generated and Bayesian posterior probabilities (BPP) were estimated.

Fungal strains were deposited at the Mycotheca Universitatis Taurinensis (MUT) collection at the University of Turin. Sequences were registered in the GenBank database (Table S3, Supplementary Materials).

### 2.3. Small-Scale Fermentation and OSMAC Approach 

#### 2.3.1. Multiwell Culture and Co-Culture Conditions

The isolated fungi were screened for the production of secondary metabolites by applying the OSMAC approach, with the exception of *Tetracladium* sp. that did not grow in axenic culture. Fungi were pre-grown in Petri dishes (9 cm Ø) containing Wickerham’s medium (WH; yeast extract 3 g, malt extract 3 g, peptone 5 g, glucose 10 g, agar 15 g, Sigma-Aldrich, Saint Louis, USA, up to 1 L of DI H_2_O, pH adjusted to 7.2–7.4) and incubated in the dark at 24 °C for seven days.

Subsequently, 24 multiwell plates were prepared with 1.5 mL/well of the following solid media: WH, WHS (WH added with 3% NaCl), Potato Dextrose Agar (PDA; potato extract 4 g, dextrose 20 g, agar 15 g, up to 1 L DI H_2_O) PDAS (PDA added with 3% NaCl), Gelatin Agar (GA; gelatin 20 g, agar 15 g, up to 1 L DI H_2_O), and GAS (GA added with 3% NaCl). Four replicates for each medium were set up.

Two cultural lines were set: (1) pure fungal cultures of the tested strains were inoculated into the multiwell plates by placing 4 mm Ø mycelium discs (from the margin of actively growing colonies) in the center of each well; (2) co-culture lines were prepared by inoculating a smear of *Streptomyces* sp. MUT 2498 on the opposite side of the pure fungal culture discs. This bacterial strain, previously isolated from the Atlantic sponge *Dysidea fragilis*, was chosen due to its ability to grow in all the tested conditions.

Inoculated multiwell plates were incubated in the dark at 24 °C for two weeks. The production of exudates (***e***), soluble pigments (***s***), and production of conidia (***h***) were recorded. Fungal growth was classified as follows: no growth (−), development of the fungal mycelium only on the inoculum disc (+), mycelium covering of the area of the well (++), and fungal mycelium covering the entire surface of the well (++).

Interactions with *Streptomyces* sp. MUT 2498 were determined as antagonism (***a***) where the growth of the fungus stopped at a distance from the bacterial colony, predominance (***p***) where the fungus grew on the bacterium, and no growth of the bacterium (***n***) [27].

#### 2.3.2. Extraction Procedure and Chemical Analyses

For each well, the fungal biomasses and their associated media were freeze-dried. Dried samples were suspended in 50 mL of MeOH:CH_2_Cl_2_ (1:1, *v*/*v*) and homogenized with an Ultra turrax IKA device. The samples were then extracted three times by sonication with 50 mL of MeOH:CH_2_Cl_2_ (1:1, *v*/*v*) and filtered. The combined filtrates were evaporated under reduced pressure.

In order to obtain the chemical fingerprint of each fungus (tested under different conditions), the crude extracts were re-suspended in a mixture of MeOH:CH_2_Cl_2_ (1:1, *v*/*v*) to reach a final concentration of 10 mg/mL. HPLC analyses were performed with a Waters Alliance 2695 HPLC system (Waters Corporation, Milford, MA) coupled with a Waters 996 photodiode array detector, using a Macherey-Nagel NUCLEODUR^®^ Sphinx column (250 × 4.6 mm, 5 μm). The mobile phase was composed of H_2_O (plus 0.1% HCO_2_H) and acetonitrile (CH_3_CN; plus 0.1% HCO_2_H), with the following gradient: H_2_O:CH_3_CN 90:10 to 0:100 for 30 min, 0:100 for 5 min, and 0:100 to 90:10 for 15 min. 

Change in chemical diversity was evaluated on the basis of the presence of absorbance peaks at a selected wavelength (280 nm) between 5 and 35 min of retention time. The following classes were attributed: 0–3 peaks (class 1), 4–7 peaks (class 2), 8–11 peaks (class 3), and ≥12 peaks (class 4).

### 2.4. Scale-Up of *Eurotium chevalieri* MUT 2316

The test performed in multiwell plates recognized the fungus *E. chevalieri* MUT 2316 on PDAS as the richest in secondary metabolites. Therefore, this strain was selected for further studies; both solid and liquid culture conditions were assessed. 

#### 2.4.1. Solid Media Culture Condition and Secondary Metabolite Extraction from *Eurotium chevalieri* MUT 2316

*E. chevalieri* MUT 2316 was pre-grown on PDA, and mycelium discs (4 mm Ø) were taken from the margin of actively growing colonies to inoculate onto 200 Petri dishes (6 cm Ø) with PDAS. Plates were incubated for two weeks at 24 °C. Petri dishes were then lyophilized to obtain 88 g of freeze-dried material. The sample was suspended in a mixture of MeOH:CH_2_Cl_2_ (1:1, *v*/*v*) and homogenized with an Ultra turrax IKA device. Secondary metabolites were firstly extracted 10 times with 2.5 L of EtOAc:CH_2_Cl_2_ (1:1, *v*/*v*) and the fungal biomass was filtered under reduced pressure. The second extraction was performed (12 times) with 3 L (total) of MeOH:CH_2_Cl_2_ (1:1, *v*/*v*) and the sample was filtered under vacuum. The two organic filtrates were evaporated under reduced pressure to yield orange-red organic extracts for both EtOAc:CH_2_Cl_2_ (1.9 g) and MeOH:CH_2_Cl_2_ (4.6 g) fractions.

The EtOAc:CH_2_Cl_2_ organic fraction was further processed and fractionated by RP-C18 flash chromatography (elution with a decreasing polarity gradient of H_2_O/MeOH from 1:0 to 0:1, then MeOH/CH_2_Cl_2_ from 1:0 to 0:1). The first MeOH fraction (1.1 g), which was the richest in secondary metabolites, was further purified by semi-preparative normal-phase HPLC (Lichrosorb Diol, 250 × 10 mm, 5 µm, gradient: cyclohexane (Cy):EtOAc 90:10 to 0:100 for 30 min, 0:100 for 5 min, 0:100 to and 90:10 for 10 min, with a flow rate of 3 mL/min) to afford pure echinulin (**1**) (3.2 mg), neoechinulin A (**2**) (3.3 mg), dihydroauroglaucin (**4**) (4.3 mg), flavoglaucin (**5**) (11.2 mg), isodihydroauroglaucin (**6**) (1.5 mg), and neoechinulin D (**7**) (1.5 mg).

In order to increase the yield of the fractions/compounds obtained, the original MeOH fraction was subsequently fractionated by diol silica gel chromatography with a gradient from 100% Cy to 100% EtOAc, to afford physcion (**3**) (9.0 mg) and several fractions. Two fractions were further purified by semi-preparative reversed-phase HPLC (Macherey-Nagel NUCLEODUR® Sphinx RP, 250 × 10 mm, 5 µm, gradient H_2_O/CH_3_CN plus 0.1% HCO_2_H 90:10 to 0:100 for 30 min, 0:100 for 5 min, and 0:100 to 90:10 for 10 min, with a flow rate of 3 mL/min) to afford pure echinulin (**1**) (6.4 mg), neoechinulin A (**2**) (2.9 mg), dihydroauroglaucin (**4**) (7.7 mg), and flavoglaucin (**5**) (16.8 mg).

Structures of the isolated metabolites were elucidated through extensive MS and NMR spectral analyses (Figures S1–S14, Supplementary Materials), as well as comparison of their spectral data to those reported in the literature [20,28].

Stock solutions of the compounds were prepared at a concentration of 10 mg/mL (1 mg/mL for (**3**)) in dimethyl sulfoxide (DMSO) for the bioassays.

#### 2.4.2. Liquid Media Culture Condition and Secondary Metabolite Extraction from *Eurotium chevalieri* MUT 2316

Ten agar plugs of *E. chevalieri* MUT 2316 were used to inoculate 20 Erlenmeyer flasks containing 180 mL of Potato Dextrose Broth (PDB; potato extract 4 g, dextrose 20 g, Sigma-Aldrich, Saint Louis, USA, up to 1 L DI H_2_O), supplemented with 3% NaCl. Flasks were incubated for 14 days in the dark at 24 °C and 120 rpm. Cultures were filtered to separate the fungal biomass from the broth. The biomass was freeze-dried and extracted as mentioned above for the solid extraction of *E. chevalieri*. Briefly, 1 L of EtOAc:CH_2_Cl_2_ (1:1 *v*/*v*) and 1.5 L of MeOH:CH_2_Cl_2_ (1:1 *v*/*v*) were used to obtain, after evaporation under reduced pressure, 2.4 g and 2.2 g of reddish organic extracts, respectively.

The broth was extracted with 6 L of EtOAc, and the resulting EtOAc solution was evaporated under reduced pressure to give 0.8 g of a yellow-orange organic extract. The remaining aqueous fraction was fractionated by RP-C18 silica gel column and eluted with MeOH to yield, once evaporated, 2.1 g of a red-brown organic extract.

The EtOAc fraction was purified by semi-preparative reversed-phase HPLC (Macherey-Nagel NUCLEODUR^®^ Sphinx RP, 250 × 10 mm, 5 µm, gradient H_2_O/CH_3_CN (plus 0.1% HCO_2_H) 80:20 to 50:50 for 30 min, 0:100 for 5 min, and 0:100 to 80:20 for 10 min, with a flow rate of 3 mL/min) to afford pure asperflavin (**8**) (1.0 mg), cinnalutein (**9**) (1.8 mg), and cyclo-L-Trp-L-Ala (**10**) (2.8 mg) (Figures S15–S20, Supplementary Materials). 

Stock solutions of the compounds were prepared at 10 mg/mL in DMSO for the bioassays.

### 2.5. High-Resolution Mass Spectrometry Analysis and Molecular Networking Analyses

High-resolution tandem mass spectrometry (HR-MS/MS) was carried out using a Finnigan LTQ Orbitrap coupled to a Surveyor Plus HPLC pump and autosampler (Thermo Fisher, Bremen, Germany) in positive ionization mode using an MS range of *m/z* 100–2000, an MS2 range of *m/z* 200–1500, and an MSn range of *m/z* 0–1000, with 30,000 resolution. LC-MS data were acquired using Xcalibur version 2.2. HR-MS/MS raw data files were converted from .RAW to .mzXML format using the Trans-Proteomic pipeline (Institute for Systems biology, Seattle) [29], and clustered with MS-Cluster using Global Natural Products Social Molecular Networking (GNPS-https://gnps.ucsd.edu) [30]. A molecular network was created using the online workflow at GNPS. The following settings were used for generation of the network: minimum pairs cos 0.6; parent mass tolerance, 1.0 Da; ion tolerance, 0.2; network topK, 10; minimum matched peaks, 6; minimum cluster size, 2. Data were visualized and analyzed using Cytoscape 3.6.0.

The compounds common to both liquid and solid growth conditions of *E. chevalieri* MUT 2316 were highlighted, as well as the compounds unique to each condition.

### 2.6. Antiviral Assays

#### 2.6.1. Cells and Viruses

African green monkey kidney cells (VERO, ATCC-American Type Culture Collection CCL-81) and Madin Darby Canine Kidney cells (MDCK, ATCC^®^ CRL-2936™) were propagated in Dulbecco’s modified Eagle medium (DMEM; Euroclone), supplemented with 10% fetal bovine serum (FBS; Euroclone), 2 mM l-glutamine, 1 mM sodium pyruvate, 100 U/mL penicillin, and 100 μg/mL streptomycin sulfate.

Acyclovir-sensitive herpes simplex virus 1 (HSV-1) clinical isolate (kindly provided by V. Ghisetti, Amedeo di Savoia Hospital, Turin, Italy) was propagated and titrated by plaque assay on VERO cells as described previously [31]. The influenza A virus strain A/Puerto Rico/8/34 (IAV) was a generous gift from Arianna Loregian (University of Padua, Italy), and was propagated and titrated by plaque assay on MDCK cells. Infections with IAV were performed in the presence of 2 µg/mL TosylPhenylalanylChloromethylKetone -treated trypsin from bovine pancreas (Sigma-Aldrich, Saint Louis, USA) and 0.14% bovine serum albumin (BSA) (Sigma-Aldrich, Saint Louis, USA) in serum-free complete medium [32].

#### 2.6.2. Cytotoxicity Assays

To determine cell viability, confluent MDCK or VERO cells were seeded in 96-multiwell plates and exposed to increasing concentrations of the compounds (12.5 µg/mL, 25.0 µg/mL, or 50.0 µg/mL), with the exception of physcion (**3**), for which lower concentrations (6.25 µg/mL, 12.5 µg/mL, 25.0 µg/mL) were used to avoid DMSO toxicity. The plates were incubated for 48 h at 37 °C, and then 100 μL of CellTiter-Glo reagent (Promega) was added to lyse the cells and to determine cell viability according to the producer’s instructions. The compounds were assessed for their antiviral activity at the concentrations that produced at least 70% cell viability.

#### 2.6.3. Plaque Reduction Assay

For plaque reduction assays (PRA) with IAV and HSV-1 viruses, MDCK and VERO cells were seeded in 24-well plates at a density of 3 × 10^5^ or 6 × 10^4^ cells per well, respectively. The next day, cultures were treated with the selected compounds at different concentrations 1 h prior to infection, and then infected with 35 plaque-forming units (PFU)/well of either HSV-1 or IAV in the presence of the compounds. Following virus adsorption (1 h at 37 °C for IAV or 2 h at 37 °C for HSV-1), cultures were maintained in medium containing the corresponding compounds, supplemented with 0.14% BSA, 2 µg/mL trypsin, and 0.7% Avicel for IAV, or with 3% FBS and 0.9% Avicel for HSV-1. All compounds’ concentrations were tested in duplicate in two independent experiments. Control wells with mock-infected cells and untreated virus-infected cells were included in each plate. At 48 h post-infection (p.i.), cell monolayers were fixed with 4% formaldehyde for 1 h at room temperature (RT) and stained with a solution of 1% crystal violet for 40 min, before viral plaques were microscopically counted. The mean plaque counts for each compound were expressed as a percentage of the mean plaque count for the control virus.

### 2.7. Antibacterial Assays

#### 2.7.1. Bacterial Growth Conditions

Representatives of Gram-positive and Gram-negative bacteria were selected for the antimicrobial susceptibility tests. Three Gram-positive reference strains (*Staphylococcus aureus* ATCC 29213, *Streptococcus pneumoniae* ATCC 49619, and *Enterococcus faecalis* ATCC 29212) and two Gram-negative reference strains (*Escherichia coli* ATCC 25922 and *Pseudomonas aeruginosa* ATCC 27853) were used. Furthermore, antibacterial activity of the isolated compounds was also assessed against recently isolated clinical strains, selected and characterized as resistant or multidrug-resistant (MDR), from the MicroMiB collection owned and maintained by the Laboratory of Clinical Microbiology and Virology of the University of Milano-Bicocca, Monza, Italy: a methicillin-resistant *Staphylococcus aureus* (MRSA) Monza-PFI, a fluoroquinolone-resistant *S. aureus* Monza-FD1, and a macrolide-resistant *S. pneumoniae* Monza-82. MDR strains are conventionally defined as strains that are resistant against antibiotics representative of at least three different antibiotic classes.

The strains, stored at −80 °C, were inoculated in fresh cation-adjusted Mueller–Hinton Broth (CAMHB; acid digest of casein 17.5 g, soluble starch 1.5 g, beef extract 2 g, Sigma-Aldrich, Saint Louis, USA, up to 1 L of DI H_2_O) and incubated overnight at 37 °C. *S. pneumoniae* Monza-82 was inoculated in fresh Brain Heart Infusion broth (BHI; heart infusion 5 g, calf brains 12.5 g, Na_2_HPO_4_ 2.5 g, d(+)-glucose 2 g, peptone 10 g, NaCl 5 g, Sigma-Aldrich, Saint Louis, USA, up to 1 L of DI H_2_O) with the addition of 5% defibrinated sheep blood and incubated overnight at 37 °C in 5% CO_2_ atmosphere for optimal growth.

#### 2.7.2. Minimal Inhibitory Concentration (MIC) Determination

MICs were determined following Clinical and Laboratory Standards Institute (CLSI) guidelines for the broth microdilution method [33]. Briefly, two-fold serial dilutions of the stock solutions of the tested compounds and antibiotic were prepared in cation-adjusted Mueller–Hinton Broth (CAMHB) or CAMHB + 5% defibrinated sheep blood (for *S. pneumoniae*) in 96-multiwell microtiter plates; the bacterial inoculum adjusted to 1 × 10^6^ colony-forming units (CFU)/mL was equally added to the wells to reach a final concentration of 5 × 10^5^ CFU/mL. Gentamicin, ciprofloxacin, tetracycline, erythromycin, and fusidic acid (Sigma-Aldrich, Saint Louis, USA) were used as reference antibiotics. 

The plates were incubated at 37 °C for 18–24 h. After the incubation, each well was checked for the absence of bacterial pellets and the clearness of the solution; then, the MIC value (μg/mL), defined as the lowest concentration of antimicrobial agent causing visible inhibition of bacterial growth, was determined.

All the tested compounds, except for antibiotics, were solubilized in 100% DMSO (stock solutions) and appropriate dilutions were set up to prevent solvent toxicity. Positive strain controls without the antimicrobial compounds, controls with DMSO, and non-inoculated media were run in parallel with the tested compounds as internal controls.

#### 2.7.3. Minimal Bactericidal Concentration (MBC) Determination

The MBC was determined after the MIC assays; briefly, once the MIC was determined, the content of those wells and the wells with 2× MIC and 4× MIC concentration were plated on Tryptic Soy Agar (TSA; pancreatic digest of casein 15 g, peptic digest of soybean meal 5 g, NaCl 5 g, agar 15 g, Sigma-Aldrich, Saint Louis, USA, up to 1 L of DI H_2_O) or Mueller–Hinton Fastidius Agar (MHF; meat extract 2 g, acid hydrolysate of casein 17.5 g, starch 1.5 g, agar 17 g, horse blood mechanically defibrinated 5%, β-NAD 0.02 g) for the *Streptococcus* strains. Plates were incubated at 37 °C in aerobic conditions or in 5% CO_2_ atmosphere and checked for bacterial growth after 24 h. The MBC values, defined as the lowest concentration of antimicrobial agent needed to kill bacteria, were determined by evaluating the minimal concentration at which an absence of bacterial growth in the agar plates was detected.

## 3. Results and Discussion

### 3.1. The Mycobiota of *Grantia compressa*

The culturable mycobiota associated with *G. compressa* were evaluated for the first time in this study. Twenty-one strains ascribable to 17 taxa were detected (Table 1).

The use of two isolation techniques, four media, and two incubation temperatures facilitated an increase in the number of cultivable fungi, considering that 70% of the fungi were found in just one of the tested conditions (Table 1).

Overall, the mycobiota of *G. compressa* were composed of Ascomycota (77.8%) and Basidiomycota (22.2%). The dominance of Ascomycota is confirmed in many marine environments [14,53]. The high percentage of Basidiomycota is surprising given that several studies indicated the low abundance of Basidiomycota in sponges (lower than 10% [18,54,66,67] or null [47,56,68,69,70,71]).

A low rank identification of taxa is essential for the de-replication process useful for the discovery of new compounds [72,73]. By means of a polyphasic approach, 86% of taxa were identified at species level and 14% at genus level. A high percentage of taxa (41%) remained sterile in axenic culture; this is not unusual for marine fungi [47,53,68]. Sterile mycelia included five Basidiomycota that were identified by Poli et al. [74], while the doubtful systematic position of two Ascomycota, namely MUT 2273 and MUT 2274, was clarified by systematic analysis and they were identified as *Emericellopsis pallida* (Figure S21, Supplementary Materials).

Overall, while *Ceriporia lacerata*, *Penicillium paneum*, and *Rhodotorula mucilaginosa* are reported here for the first time in association with marine sponges, for *Psathyrella candolleana*, it is the first report from the marine environment. Interestingly, Zhao et al. [34] found a strain of *C. lacerata*, capable of producing novel secondary metabolites, in association with another marine invertebrate, the crown-of-thorns starfish *Acanthaster planci*.

*G. compressa*, was sampled along the Irish coast with three additional sponges (*Dysidea fragilis, Pachymatisma johnstonia*, and *Sycon ciliatum*) that possessed four common fungal species in their mycobiota [18]. Intriguingly, three out of the four fungal species, namely *Cladosporium allicinum, C. cladosporioides*, and *Tolypocladium cylindrosporum*, were also isolated from *G. compressa*, supporting the hypothesis that fungal species retrieved from different sponge species may belong to a transient mycobiota, probably not strictly associated with the animals [67]. Compared to the three other sponges mentioned above, *G. compressa* hosted the lowest fungal diversity. However, the taxa recorded (17) are in line with other studies on sponge-associated mycobiota: for instance, 19 fungal genotypes were identified in the Atlantic sponge *Haliclona simulans* [75], and a pool of several sponge species allowed the description of about 20 fungal taxa [43,48]. Several factors, such as isolation techniques, sponge tissue structure, and the ability of the sponge to produce metabolites, may influence the number of fungal taxa retrieved [47]. Concerning the capability of *G. compressa* to select the fungal community by producing particular substances, there are no reports about the metabolome of this sponge. However, since also the bacterial diversity (12 strains) was particularly low, as previously reported [76], this sponge might be able to control its microbial load.

### 3.2. OSMAC Approach

#### 3.2.1. Effect of Media on Fungal Development and Chemical Fingerprint

**Figure 1:**
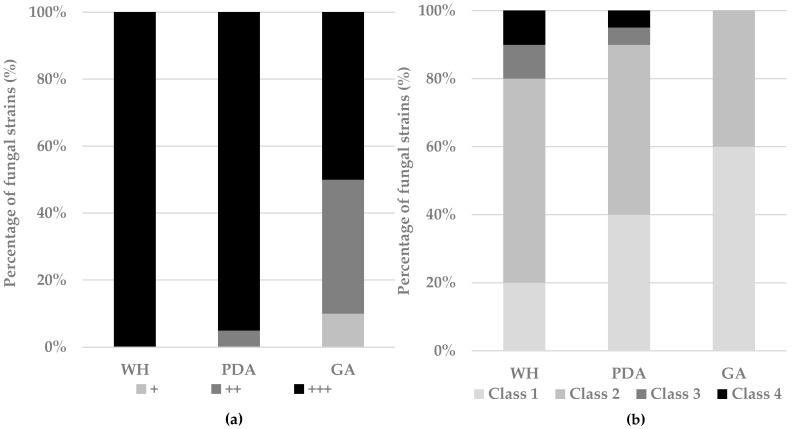
(**a**) Fungal growth on three media, classified as development of the fungus on the inoculum (+), occupation of half the area of the well (++), or complete growth of the fungus on all the surface of the well (+++). (**b**) Change in the chemical diversity of fungal extracts due to the different conditions assessed. Classification based on the number of HPLC ultraviolet (UV) 280-nm peaks: 0–3 peaks (class 1), 4–7 peaks (class 2), 8–11 peaks (class 3), and ≥12 peaks (class 4).

Multiwell assays to test different growth conditions are considered a valuable method for the screening of marine-derived fungi as producers of interesting metabolites [8,77]. The optimal growth of fungi was the first parameter that was taken into account; indeed, in light of industrial exploitation for secondary metabolite production, a fast-growing fungus must be preferred. Among the culture media tested, WH medium supported fungal growth of all the strains; after 14 days, all fungi occupied the entire medium surface (Figure 1a). PDA also supported the fungal growth and 95% of the fungi covered the entire medium surface (Figure 1a). These results are in line with a previous work that demonstrated that two weeks are necessary for slow-growing species to cover the entire surface of a 24-well plate [78]. From a chemical point of view, nutrient-rich media better induce metabolic diversity (Figure 1b). Indeed, classes 3 and 4 (high metabolic production), accounted for 10% and 20% of the tested strains on PDA and WH, respectively. On the contrary, the low-nutrient gelatin-based medium (GA) did not support proper fungal growth; only 50% of fungi occupied the entire well on GA after two weeks (Figure 1a). This affected the metabolic diversity that was strongly reduced and ascribable only to classes 1 and 2 (Figure 1b).

The OSMAC approach was proven to be a powerful method. In fact, each of the tested fungi showed different metabolic classes in at least one of the three media tested, thus underlining the best conditions for the stimulation of secondary metabolites (Table S4, Supplementary Materials). The only exceptions were represented by *C. allicinum* MUT 2307 and *C. cladosporioides* MUT 2315, showing the same metabolic class (2) in all three conditions, although the occurrence of exclusive peaks characterized both WH and PDA (Table S4, Supplementary Materials).

#### 3.2.2. Effect of Salt on Fungal Development and Chemical Fingerprint

**Figure 2:**
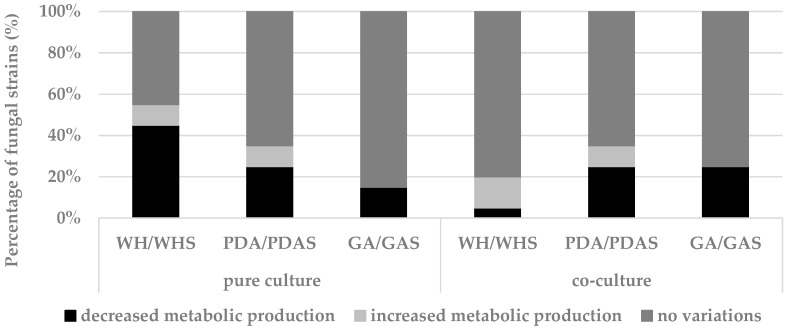
Percentage of fungal strains in which the presence of salt increased, decreased, or did not influence the metabolic diversity (same metabolic classes), related to the different conditions tested (three media in pure and co-culture).

The presence of salt did not affect the number of secondary metabolites detected for most fungi under different conditions (Figure 2). The metabolism of the remaining fungi was mostly inhibited by the presence of salt (Figure 2), i.e., on WHS, for three out of five Basidiomycota, namely *C. lacerata* MUT 2288, *Coprinellus* sp. MUT 2332, and *P. candolleana* MUT 2331 (Tables S4 and S5, Supplementary Materials), the production of secondary metabolites and the growth rate decreased in the presence of salt. On the contrary, a small number of fungi produced more metabolites in the presence of salt, i.e., *Penicillium chrysogenum* MUT 2321 on both PDAS and WHS (Table S4, Supplementary Materials).

Overall, the different abilities of fungi to tolerate salt could be related to their position within sponges. Endophytic fungi, not exposed to osmotic pressure, were already detected within sponges [79] and, consequently, some of the isolated fungi found within sponge tissues could be negatively affected by the presence of salt. Certainly, salt can be a stress factor for endophytes, and it can also mimic environmental conditions for sponge-associated fungi more exposed to seawater, modulating their metabolic production as already demonstrated [80,81,82].

#### 3.2.3. Effect of Co-Culture on Fungal Development and Chemical Fingerprint 

**Figure 3:**
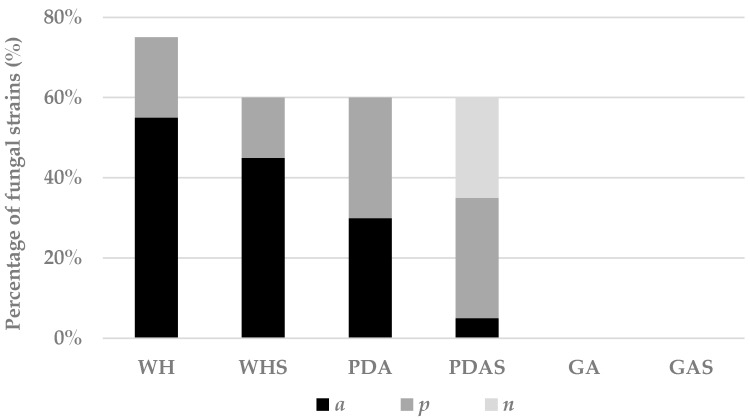
Percentage of fungi interacting in different ways with the bacterium. Interactions are classified as antagonism (***a***), predominance (***p***), and total inhibition of the bacterial growth (***n***) on the six tested media.

The co-culture differentially influenced fungal growth (Table S5, Supplementary Materials) possibly due to space and nutrient competition that generally occur in dual culture [83]. On PDAS, 25% of the fungi completely inhibited the bacterium (Figure 3); this was probably due to the slow bacterial growth on this medium compared to the other media. Overall, on WH, WHS, PDA, and PDAS, 75%, 60%, 60%, and 35% of the fungi, respectively, interacted with the bacterium with an antagonistic response or over-growing it (Figure 3). When an antagonistic response (***a)*** or dominance of the fungus (***p***) was observed, the metabolic production respectively increased in 15% and 16% of the cases (Tables S4 and S5, Supplementary Materials). Noteworthy, even when the number of metabolites was constant or decreased, exclusive peaks were found in one-third of the fungi; this was the case of *E. pallida* MUT 2273 that clearly showed an antagonistic response against the bacterium on WH and PDA and produced exclusive peaks not detected in other conditions (Tables S4 and S5, Supplementary Materials). The presence of exclusive peaks in co-culture represents direct evidence of the interaction on a specific medium between the two organisms tested; however, whether the fungus or the bacterium is responsible for the production of the compounds was difficult to determine.

#### 3.2.4. General Remarks on the OSMAC Approach

**Figure 4:**
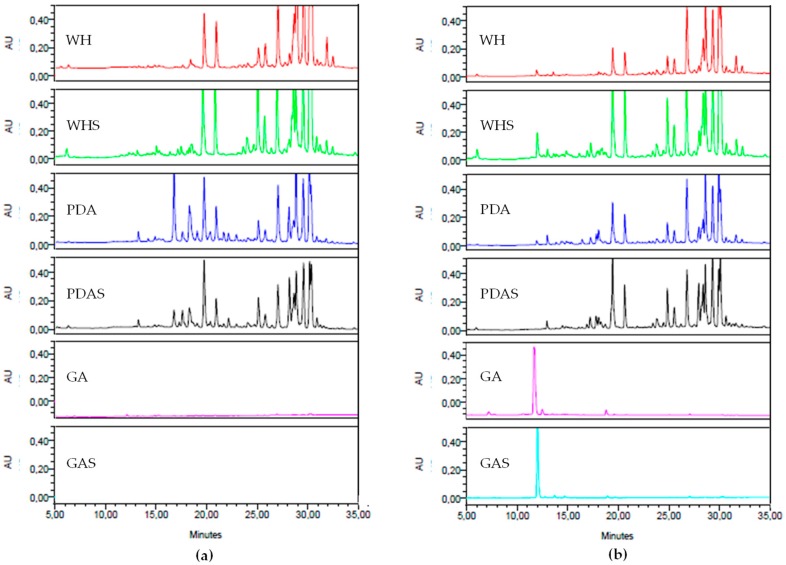
Comparative analysis of the HPLC chemical fingerprints (λ = 280 nm) of the crude organic extracts of *Eurotium chevallieri* MUT 2316 after 14 days of incubation on six different media (Wickerham’s (WH), WH with salt (WHS), Potato Dextrose Agar (PDA), PDA with salt (PDAS), Gelatin Agar (GA), and GA with salt (GAS)) in (**a**) pure culture and (**b**) co-culture. Intensity was fixed at 0.5 AU for all samples.

Considering the 12 conditions tested, it was clear that some fungi presented a reduced metabolic diversity, which was not particularly influenced by growth conditions. This was the case for *C. lacerata* MUT 2288, *Coprinellus* sp. MUT 2282, and *Euthypella scoparia* MUT 2334 (Table S4, Supplementary Materials). On the contrary, *E. chevalieri* MUT 2316 produced more metabolites than any other tested fungi, in particular on all nutrient-rich media in pure culture and in co-culture (Figure 4).

Moreover, the production of secondary metabolites appeared to be specific for each strain (Table S4, Supplementary Materials). This was very evident for *T. cylindrosporum* (MUT 2410 and MUT 2413) and *E. pallida* (MUT 2273 and MUT 2274), while it was not for *C. allicinum* (MUT 2307 and MUT 2313), *P. paneum* (MUT 2322 and MUT 2326), and *Coprinellus* sp. (MUT 2282 and MUT 2332). 

Overall, the OSMAC approach is a valuable method for the induction of different secondary metabolites [8]. Several fungi showed enhanced metabolic production/diversity, together with a clear morphological variation. This might be useful for a first screening of the most promising culture condition; usually, when clear morphological variations are observed, the fungus produces different metabolites.

### 3.3. *Eurotium chevalieri* MUT 2316: Molecular Networking and Compounds

*E. chevalieri* MUT 2316 produced a higher number of and more chemically diverse metabolites in comparison to the other fungi tested. For this reason, *E. chevalieri* MUT 2316 was selected for scale-up and metabolite identification. The medium that supported better growth and chemical diversity of the fungus was the medium PDAS. Both solid and liquid culture conditions were tested for the scale-up of *E. chevalieri* MUT 2316.

The growth of *E. chevalieri* in solid medium yielded 628 parent ions, half of which were common to the same fungus grown in liquid media, which yielded 536 compounds.

**Figure 5:**
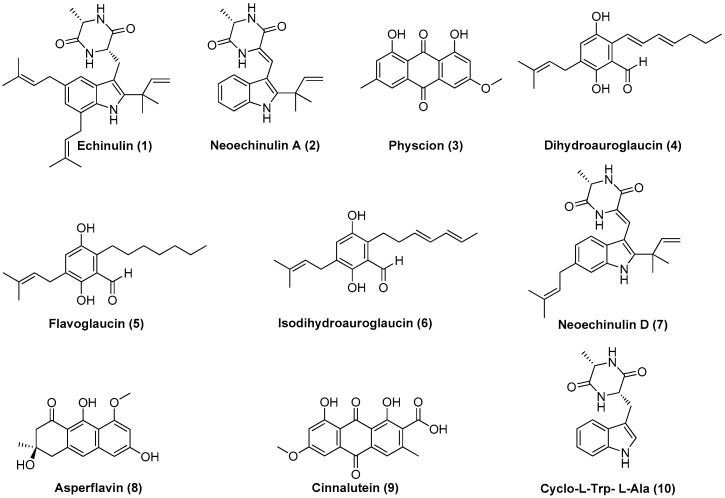
Structures of **1**–**10**.

The purification process of the crude organic extracts from the solid and liquid cultures yielded 10 compounds (Figure 5): echinulin (**1**), neoechinulin A (**2**), physcion (**3**), dihydroauroglaucin (**4**), flavoglaucin (**5**), isodihydroauroglaucin (**6**), neoechinulin D (**7**), asperflavin (**8**), cinnalutein (**9**), and cyclo-l-Trp-l-Ala (**10**).

**Figure 6:**
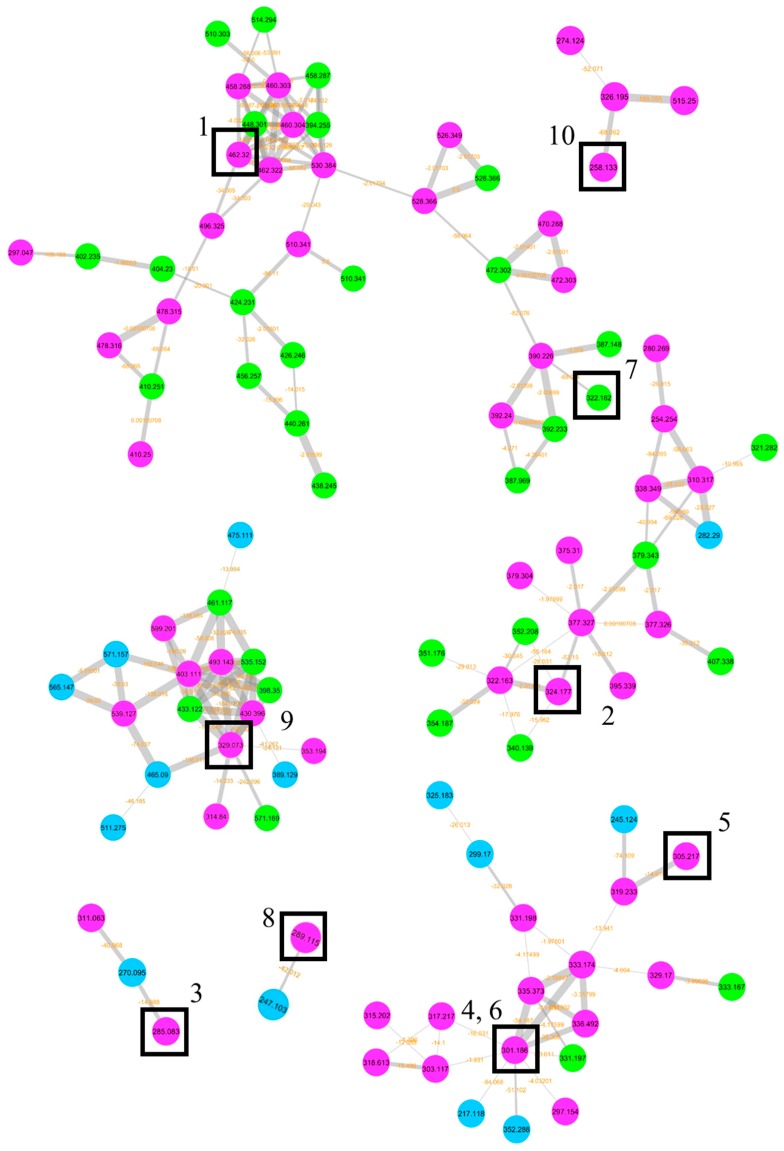
Molecular network constructed using MS/MS data from the crude organic extracts originating from the solid and liquid culture conditions. The clusters of the pure compounds obtained in this study are reported for echinulin (**1**), neoechinulin A (**2**), physcion (**3**), dihydroauroglaucin (**4**), flavoglaucin (**5**), isodihydroauroglaucin (**6**), neoechinulin D (**7**), asperflavin (**8**), cinnalutein (**9**), and cyclo-l-Trp-l-Ala (**10**). Colors highlight the compounds only detected in solid culture condition (green), in liquid culture condition (blue), and in both (fuchsia).

The graphical representation of the molecular network allowed underlining in which condition (solid/liquid) the pure compounds isolated in this study were produced. Figure 6 shows the molecular clusters where the compounds were found; green, blue, and fuchsia nodes indicate compounds found in solid media, liquid media, and in both conditions, respectively. Nine compounds were produced in both solid and liquid culture conditions, while **7** was produced only in the solid medium. Moreover, the presence of different compounds in the same cluster underlined a common biosynthetic pathway; this was the case for **1** and **7**, as well as for **4**, **5**, and **6**.

### 3.4. *Eurotium chevalieri*-Derived Compounds Show Antiviral Activity against Herpes and Influenza Viruses

**Figure 7:**
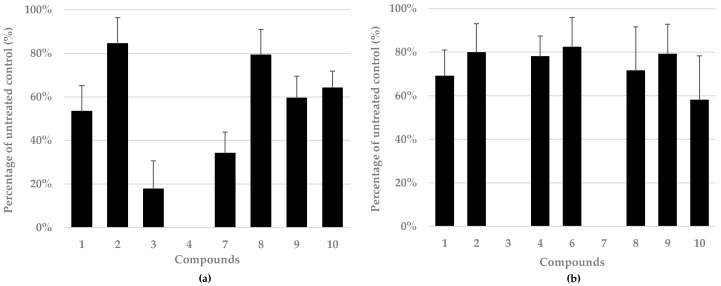
Effect of the compounds derived from *E. chevalieri* on (**a**) influenza A virus (IAV) and (**b**) herpes simplex virus 1 (HSV-1) replication. The mean plaque counts for each molecule are expressed as a percentage of the mean plaque count of the control. The data shown are the means ± standard deviations (SD) from two independent experiments performed in duplicate.

To evaluate the antiviral activity of the metabolites (**1–10**) isolated from *E. chevalieri* MUT 2316, we investigated their effects on the replication of IAV and a clinical isolate of HSV-1. Prior to the assessment of the antiviral activity by PRA, cell viability assays were performed to exclude non-specific cell toxicity of the tested compounds against MDCK and VERO cells. To this end, different concentrations of each compound were tested, and compounds were considered not toxic only if they produced at least 70% cell viability after 48 h of treatment. Based on this parameter, flavoglaucin (**5**) and isodihydroauroglaucin (**6**), even at the lowest dose (12.5 µg/mL), were considered toxic for MDCK cells, whereas only flavoglaucin (**5**) was considered toxic for VERO cells (50% cytotoxic concentration (CC_50_) for both compounds <12.5 µg/mL). Thus, flavoglaucin (**5**) and isodihydroauroglaucin (**6**) were not further investigated for their antiviral activity against IAV, while flavoglaucin (**5**) was not tested against HSV-1. Based on results obtained from the cytotoxicity assay for IAV, echinulin (**1**) and neoechinulin D (**7**) were tested at concentration of 50 µg/mL, neoechinulin A (**2**), physcion (**3**), dihydroauroglaucin (**4**), and cyclo-l-Trp-l-Ala (**10**) were tested at 25 µg/mL, and asperflavin (**8**) and cinnalutein (**9**) were tested at 12.5 µg/mL. For HSV-1, echinulin (**1**)**,** neoechinulin A (**2**)**,** cyclo-l-Trp-l-Ala (**10**)**,** neoechinulin D (**7**), and asperflavin (**8**) were examined at 50 µg/mL, physcion (**3**) and dihydroauroglaucin (**4**) were examined at 25 µg/mL, and isodihydroauroglaucin (**6**) and cinnalutein (**9**) were examined at 12.5 µg/mL. Among the compounds tested, dihydroauroglaucin (**4**), physcion (**3**), and neoechinulin D (**7**) inhibited the replication of IAV by 100%, 80%, and 70%, respectively (Figure 7a); the antiviral activity against IAV was observed for the first time for five out of eight compounds, while echinulin (**1**), neoechinulin A (**2**), and cyclo-l-Trp-l-Ala (**10**) were already tested against another strain of Influenza (A/WSN/33) by Chen et al. [84]. For HSV-1 replication (Figure 7b), total inhibition (100%) was observed for the first time for both physcion (**3**) and neoechinulin D (**7**). Activity against HSV-1 was previously assessed only for isodihydroauroglaucin (**6**) [28].

Overall, these results suggest significant antiviral activities for some secondary metabolites from *E. chevalieri*, such as physcion (**3**)**,** dihydroauroglaucin (**4**), and neoechinulin D (**7**), against two important human viral pathogens. These data indicate that the antiviral potential of these compounds deserves further analyses. In this regard, a detailed analysis of their mechanism of action against both IAV and HSV is underway.

### 3.5. *Eurotium chevalieri*-Derived Compounds Show Antibacterial Activity 

All the metabolites (**1**–**10**) were evaluated for their antibacterial activity against reference strains of Gram-positive and Gram-negative bacteria, as well as on selected clinical isolates of multidrug-resistant bacteria. Table 2 reports the MIC and MBC values of each compound. Isodihydroauroglaucin (**6**) was the most promising with antibacterial activity on the majority of the Gram-positive bacteria tested (except *S. pneumonia* ATCC 49619), with MIC values ranging from 4 µg/mL to 64 µg/mL; the lowest value was observed against the macrolide-resistant *S. pneumoniae* Monza-82. Isodihydroauroglaucin (**6**) showed similar MIC values when tested against *Bacillus cereus* and *Salmonella typhimurium* [28]. Similar to isodihydroauroglaucin (**6**), dihydroauroglaucin (**4**) was active against the majority of the Gram-positive bacteria (except for *S. pneumonia* ATCC 49619), with MIC values of 128 µg/mL, 64 µg/mL, and 8 µg/mL on *S. aureus* strains, *E. faecalis*, and *S. pneumoniae*, respectively. This compound was previously considered inactive against reference and MRSA *S. aureus* strains, probably due to the low concentration tested [85]. Here, we report for the first time the antibacterial activity of dihydroauroglaucin (**4**).

Asperflavin (**8**) was active against all *S. aureus* strains and *S. pneumoniae* Monza-82, with MIC values of 64 µg/mL and 32 µg/mL, respectively. Ganihigama et al. [86] reported the antibacterial activity of asperflavin (**8**) against *Mycobacterium tuberculosis* (MIC 25 μg/mL).

This study represents the first assessment of the antibacterial activity of cinnalutein (**9**), showing positive results against the fluoroquinolone-resistant *S. aureus* Monza-FD1 (MIC 128 μg/mL) and macrolide-resistant *S. pneumoniae* Monza-82 (MIC 32 μg/mL).

Of note, *S. pneumoniae* Monza-82 showed sensitivity to all the compounds mentioned above, including physcion (**3**) and neoechinulin D (**7**).

Physcion (**3**) was previously tested against *Micrococcus luteus* (MIC 200 μg/mL), *Klebsiella pneumoniae* (MIC 250 μg/mL), and *P. aeruginosa* (MIC 200 μg/mL) [87]. On the contrary, Zin et al. [20] observed no antibacterial activity for physcion (**3**). This highlights that the use of different bacterial strains can significantly influence the results. The other compounds (Table 2) did not show antibacterial activity at the concentrations tested (0.06–128 μg/mL for **1**, **2**, and **10**; 0.015–32 μg/mL for **5**), confirming previous reports for cyclo-l-Trp-l-Ala (**10**) [88] and echinulin (**1**) [28,85,89,90].

Isodihydroauroglaucin (**6**) also showed bactericidal activity for most of the bacteria tested, whereas dihydroauroglaucin (**4**) and asperflavin (**8**) showed positive results only against the *S. pneumoniae* Monza-82 strain. 

## 4. Conclusions

In conclusion, in this study, we highlighted the high biodiversity of marine-derived fungi inhabiting the Atlantic sponge *G. compressa*, and the chemodiversity and biotechnological potential of the fungal strain *E. chevalieri* MUT 2316. The OSMAC approach, applied to all the fungi retrieved in *G. compressa*, was demonstrated to be a powerful method to stimulate the production of an incredible variety of secondary metabolites.

*E. chevalieri* MUT 2316 produced the highest number of metabolites and among the compounds isolated; dihydroauroglaucin (**4**)**,** physcion (**3**), and neoechinulin D (**7**) completely inhibited viral replication of IAV or HSV-1. Moreover, six compounds were active against Gram-positive bacteria; with isodihydroauroglaucin (**6**) active against most of the Gram-positive bacteria tested (MIC 4-64 µg/mL) with bactericidal activity. 

Finally, the results obtained in the present work demonstrate that even already known compounds can show new and promising biological activities. 

## Figures and Tables

**Table 1:** Fungal taxa isolated from *Grantia compressa* with the percentage relative abundance (RA) of recovery and the isolation conditions (isolation methods, media, and temperature). The species already found in the marine environment (MA) and associated with sponges (SP) are reported, as well as the first record (FR).

MUT CODE	Taxa	RA (%)	Isolation Method		Isolation Media				Incubation Temperature		Report	
			DP	HO	CMASW	GASW	MEA	SWA	15 °C	25 °C	MA	SP
2282	*Coprinellus* sp. *	8.7		x			x			x	-	-
2332				x	x					x		
2288	*Ceriporia lacerate* *	4.3		x			x			x	[34,35]	FR
2307	*Cladosporium allicinum*	13.0		x			x			x	[16,36]	[18]
2313				x	x				x			
2314	*Cladosporium cladosporioides*	4.3		x			x			x	[15,16,36,37,38,39,40,41,42]	[18,43,44,45,46,47,48]
2315	*Cladosporium pseudocladosporioides*	8.7		x		x			x		[16,38]	[18]
2273	*Emericellopsis pallida* *	8.7	x			x			x		[15]	[18]
2274				x		x			x			
2316	*Eurotium chevalieri*	4.3		x			x			x	[15,40,42,49,50]	[47]
2334	*Euthypella scoparia* *	4.3		x		x				x	[51]	[8]
2317	*Fusarium solani*	4.3		x		x				x	[52,53]	[54,55]
2321	*Penicillium chrysogenum*	4.3		x	x				x		[15,38,39,42]	[47,54,56,57]
2328	*Penicillium oxalicum*	4.3		x		x			x		[15,42,53,58,59,60]	[61]
2322	*Penicillium paneum*	8.7		x		x				x	[15,62]	FR
2326				x			x			x		
2331	*Psathyrella candolleana* *	4.3		x	x					x	FR	FR
2415	*Rhodotorula mucilaginosa*	4.3	x		x					x	[15,63,64]	FR
-	*Tetracladium* sp. *	4.3		x	x					x	-	-
2410	*Tolypocladium cylindrosporum*	8.7	x					x	x		[39,53,65]	[18]
2413				x	x					x		

* Sterile mycelium. DP—direct plating; HO—homogenization; CMASW—Corn Meal Agar sea water; GASW—Gelatin Agar sea water; MEA—Malt Extract Agar; SWA—Sea Water Agar.

**Table 2:** Minimum inhibitory concentration (MIC µg/mL) and minimum bactericidal concentration (MBC µg/mL) values of compounds (**1**–**10**) against reference strains and multidrug-resistant isolates.

Bacteria	*Staphylococcus aureus*						*Enterococcus faecalis*		*Streptococcus pneumoniae*				*Pseudomonas aeruginosa*		*Escherichia coli*	
Compounds	ATCC 29213		Monza-PFI		Monza-FD1		ATCC 29212		ATCC 49619		Monza 82		ATCC 27853		ATCC 25922	
	MIC	MBC	MIC	MBC	MIC	MBC	MIC	MBC	MIC	MBC	MIC	MBC	MIC	MBC	MIC	MBC
Echinulin (**1**)	>128	N/A	>128	N/A	>128	N/A	>128	N/A	>128	N/A	>128	N/A	>128	N/A	>128	N/A
Neoechinulin A (**2**)	>128	N/A	>128	N/A	>128	N/A	>128	N/A	>128	N/A	>128	N/A	>128	N/A	>128	N/A
Physcion (**3**)	>32	N/A	>32	N/A	>32	N/A	>32	N/A	>32	N/A	16	>32	>32	N/A	>32	N/A
Dihydroauroglaucin (**4**)	128	>128	128	>128	128	>128	64	>128	>128	N/A	8	32	>128	N/A	>128	N/A
Flavoglaucin (**5**)	>32	N/A	>32	N/A	>32	N/A	>32	N/A	>32	N/A	>32	N/A	>32	N/A	>32	N/A
Isodihydroauroglaucin (**6**)	64	128	64	128	32	64	64	>128	>128	N/A	4	16	>128	N/A	>128	N/A
Neoechinulin D (**7**)	>128	N/A	>128	N/A	>128	N/A	>128	N/A	>128	N/A	64	>128	>128	N/A	>128	N/A
Asperflavin (**8**)	64	>128	128	>128	64	>128	>128	N/A	>128	N/A	32	128	>128	N/A	>128	N/A
Cinnalutein (**9**)	>128	N/A	>128	N/A	128	>128	>128	N/A	>128	N/A	32	>128	>128	N/A	>128	N/A
Cyclo-l-Trp-l-Ala (**10**)	>128	N/A	>128	N/A	>128	N/A	>128	N/A	>128	N/A	>128	N/A	>128	N/A	>128	N/A
Gentamicin	0.5	N/D	>128	N/D	1	N/D	>128	N/D	N/D	N/D	N/D	N/D	1	N/D	0.5	N/D
Ciprofloxacin	0.25	N/D	64	N/D	8	N/D	0.25	N/D	0.5	N/D	1	N/D	0.12	N/D	≤0.06	N/D
Erythromycin	0.5	N/D	8	N/D	0.5	N/D	2	N/D	0.12	N/D	16	N/D	N/D	N/D	N/D	N/D
Tetracycline	0.12	N/D	1	N/D	1	N/D	8	N/D	0.12	N/D	≤0.06	N/D	8	N/D	1	N/D
Fusidic acid	0.12	N/D	N/D	N/D	N/D	N/D	4	N/D	4	N/D	N/D	N/D	N/D	N/D	N/D	N/D

N/D, not done; N/A, not applicable.

## Supplementary Materials

The following are available online at https://www.mdpi.com/1660-3397/17/4/220/s1. Table S1: Gene loci sequenced, primers for molecular analysis and PCR programs, Table S2: List of taxa used for the phylogenetic analysis of Emericellopsis spp. and their GenBank accession number, Table S3: List of fungal strains isolated from *G. compressa* with Mycotheca Universitatis Taurinensis (MUT) and GenBank accession number, Figure S1: ^1^H NMR spectrum of echinulin (1) in CDCl3 at 500 MHz, Figure S2: ^13^C NMR spectrum of echinulin (1) in CDCl3 at 125 MHz, Figure S3:^1^H NMR spectrum of neoechinulin A (2) in CDCl3 at 500 MHz, Figure S4: ^13^C NMR spectrum of neoechinulin A (2) in CDCl3 at 125 MHz, Figure S5: ^1^H NMR spectrum of physcion (3) in CDCl3 at 500 MHz, Figure S6: ^13^C NMR spectrum of physcion (3) in CDCl3 at 125 MHz, Figure S7: ^1^H NMR spectrum of dihydroauroglaucin (4) in CDCl3 at 500 MHz, Figure S8: ^13^C NMR spectrum of dihydroauroglaucin (4) in CDCl3 at 125 MHz, Figure S9: ^1^H NMR spectrum of flavoglaucin (5) in CDCl3 at 500 MHz, Figure S10: ^13^C NMR spectrum of flavoglaucin (5) in CDCl3 at 125 MHz, Figure S11: ^1^H NMR spectrum of isodihydroauroglaucin (6) in CDCl3 at 500 MHz; Figure S12: ^13^C NMR spectrum of isodihydroauroglaucin (6) in CDCl3 at 125 MHz, Figure S13: ^1^H NMR spectrum of neoechinulin D (7) in CDCl3 at 500 MHz, Figure S14: ^13^C NMR spectrum of neoechinulin D (7) in CDCl3 at 125 MHz, Figure S15: ^1^H NMR spectrum of asperflavin (8) in CD3OD at 500 MHz, Figure S16: ^13^C NMR spectrum of asperflavin (8) in CD3OD at 125 MHz, Figure S17: ^1^H NMR spectrum of cinnalutein (9) in CD3OD at 500 MHz, Figure S18: ^13^C NMR spectrum of cinnalutein (9) in CD3OD at 125 MHz, Figure S19: ^1^H NMR spectrum of cyclo-L-Trp-L-Ala (10) in CD3OD at 500 MHz, Figure S20: ^13^C NMR spectrum of cyclo-L-Trp- L-Ala (10) in CD3OD at 125 MHz, Figure S21: Bayesian phylogram of the genus Emericellopsis based on a combined dataset of ITS and beta tubulin partial sequences. MUT 2273 and MUT 2274 were identified as *Emericellopsis pallida*. Branch numbers indicate BPP values, Table S4: Effect of different growth conditions on the HPLC-UV 280 nm chemical fingerprint of fungi, expressed as classes 1, 2, 3 and 4 at the increasing of the metabolic diversity. Exclusive peaks of each condition indicated in parenthesis. The changing in colours from dark to light blue and white indicated the increasing of the metabolic diversity, Table S5: Effect of different growth conditions on the development of the fungus (-, +, ++, +++); production of exudates (e), soluble pigments (s) and high sporulation (h); fungal-bacteria interaction as antagonistic response (a), predominance of the fungus (p) or complete inhibition of the bacterial growth (n). The changing in colours from dark to light blue and white, underlined the increasing of growth classes.
