# Supplementary material for: Marine Fungi from the Sponge Grantia compressa: Biodiversity, Chemodiversity, and Biotechnological Potential
Source: Mar Drugs. 2019 Apr 11;17(4):220. doi: 10.3390/md17040220 (PMC6520677; doi:10.3390/md17040220)
Supplement: Supplementary file 1 [file marinedrugs-17-00220-s001.pdf]

Supplementary material

Marine fungi from the sponge *Grantia compressa*: biodiversity, chemodiversity and biotechnological potential

Elena Bovio [1,5], Laura Garzoli [1], Anna Poli [1], Anna Luganini [2], Pietro Villa [3], Rosario Musumeci [3], Grace P. McCormack [4], Clementina E. Cocuzza [3], Giorgio Gribaudo [2], Mohamed Mehiri [5,*] and Giovanna C. Varese [1,*]

[1] Mycotheca Universitatis Taurinensis, Department of Life Sciences and Systems Biology, University of Turin, Viale Mattioli 25, 10125 Turin (Italy); elena.bovio@unito.it, laura.garzoli@unito.it, anna.poli@unito.it, cristina.varese@unito.it

[2] Laboratory of Microbiology and Virology, Department of Life Sciences and Systems Biology, University of Turin, Via Accademia Albertina 13, 10123 Turin (Italy); anna.luganini@unito.it, giorgio.gribaudo@unito.it

[3] Laboratory of Clinical Microbiology and Virology, Department of Medicine, University of Milano-Bicocca, via Cadore 48, 20900 Monza (Italy); p.villa18@campus.unimib.it, rosario.musumeci@unimib.it, clementina.cocuzza@unimib.it

[4] Zoology, Ryan Institute, School of Natural Sciences, National University of Ireland Galway, University Road, Galway (Ireland); grace.mccormack@nuigalway.ie

[5] University Nice Côte d'Azur, CNRS, Nice Institute of Chemistry, UMR 7272, Marine Natural Products Team, Nice 60103 (France); mohamed.mehiri@unice.fr

* Correspondence: mohamed.mehiri@unice.fr; Tel.: +33-492-076-154; cristina.varese@unito.it; Tel.: +39-011-670-5964

Table of Contents

**Table S1.**	Gene loci sequenced, primers for molecular analysis and PCR programs.
**Table S2.**	List of taxa used for the phylogenetic analysis of *Emericellopsis* spp. and their GenBank accession number
**Table S3.**	List of fungal strains isolated from *G. compressa* with Mycotheca Universitatis Taurinensis (MUT) and GenBank accession number.
**Figure S1.**	^{1}H NMR spectrum of echinulin **(1)** in $CDCl_3$ at 500 MHz.
**Figure S2.**	^{13}C NMR spectrum of echinulin **(1)** in $CDCl_3$ at 125 MHz.
**Figure S3.**	^{1}H NMR spectrum of neoechinulin A **(2)** in $CDCl_3$ at 500 MHz.
**Figure S4.**	^{13}C NMR spectrum of neoechinulin A **(2)** in $CDCl_3$ at 125 MHz.
**Figure S5.**	^{1}H NMR spectrum of physcion **(3)** in $CDCl_3$ at 500 MHz.
**Figure S6.**	^{13}C NMR spectrum of physcion **(3)** in $CDCl_3$ at 125 MHz.
**Figure S7.**	^{1}H NMR spectrum of dihydroauroglaucin **(4)** in $CDCl_3$ at 500 MHz.
**Figure S8.**	^{13}C NMR spectrum of dihydroauroglaucin **(4)** in $CDCl_3$ at 125 MHz.
**Figure S9.**	^{1}H NMR spectrum of flavoglaucin **(5)** in $CDCl_3$ at 500 MHz.
**Figure S10.**	^{13}C NMR spectrum of flavoglaucin **(5)** in $CDCl_3$ at 125 MHz.
**Figure S11.**	^{1}H NMR spectrum of isodihydroauroglaucin **(6)** in $CDCl_3$ at 500 MHz.
**Figure S12.**	^{13}C NMR spectrum of isodihydroauroglaucin **(6)** in $CDCl_3$ at 125 MHz.
**Figure S13.**	^{1}H NMR spectrum of neoechinulin D **(7)** in $CDCl_3$ at 500 MHz.
**Figure S14.**	^{13}C NMR spectrum of neoechinulin D **(7)** in $CDCl_3$ at 125 MHz.
**Figure S15.**	^{1}H NMR spectrum of asperflavin **(8)** in CD_3OD at 500 MHz.
**Figure S16.**	^{13}C NMR spectrum of asperflavin **(8)** in CD_3OD at 125 MHz.

**Figure S17.**	^{1}H NMR spectrum of cinnalutein **(9)** in CD_3OD at 500 MHz.
**Figure S18.**	^{13}C NMR spectrum of cinnalutein **(9)** in CD_3OD at 125 MHz.
**Figure S19.**	^{1}H NMR spectrum of cyclo-L-Trp- L-Ala **(10)** in CD_3OD at 500 MHz.
**Figure S20.**	^{13}C NMR spectrum of cyclo-L-Trp- L-Ala **(10)** in CD_3OD at 125 MHz.
**Figure S21.**	Bayesian phylogram of the genus *Emericellopsis* based on a combined dataset of ITS and beta tubulin partial sequences. MUT 2273 and MUT 2274 were identified as *Emericellopsis pallida*. Branch numbers indicate BPP values.
**Table S4.**	Effect of different growth conditions on the HPLC-UV 280 nm chemical fingerprint of fungi, expressed as classes 1, 2, 3 and 4 at the increasing of the metabolic diversity. Exclusive peaks of each condition indicated in parenthesis. The changing in colours from dark to light blue and white indicated the increasing of the metabolic diversity.
**Table S5.**	Effect of different growth conditions on the development of the fungus (-, +, ++, +++); production of exudates ***(e)***, soluble pigments ***(s)*** and high sporulation ***(h)***; fungal-bacteria interaction as antagonistic response ***(a)***, predominance of the fungus ***(p)*** or complete inhibition of the bacterial growth ***(n)***. The changing in colours from dark to light blue and white, underlined the increasing of growth classes.

Table S1. Gene loci sequenced, primers for molecular analysis and PCR programs.

Fungi	Gene loci and DNA regions sequenced[a]	Primers (Forward and Reverse)	PCR amplification Conditions	References for primers
Penicillium, Emericellopsis	*TUB*	BT–2a and BT–2b	94 °C: 4 min, (94 °C: 35 sec, 58 °C: 35 sec, 72 °C: 50 sec) × 35 cycles; 72 °C: 5 min	[92]
Cladosporium	*ACT*	ACT–512F and ACT–783R	94 °C: 8 min, (94 °C: 15 sec, 61 °C: 20 sec, 72 °C: 40 sec) × 35 cycles; 72 °C: 10 min	[93]
Sterile mycelia and taxa for which no specific primers are required	ITS	ITS1 and ITS4	95 °C: 5 min, (95 °C:40 s, 55 °C: 50 s, 72 °C: 50 sec) × 35 cycles; 72 °C: 8 min	[94]
Sterile mycelia	LSU	LROR and LR7	95 °C: 5 min, (95 °C: 1 min, 50 °C: 1 min, 72 °C: 2 min) × 35 cycles; 72 °C: 10 min	[95]

[a] TUB: partial beta-tubulin gene; ACT: partial actin gene; ITS: internal transcribed spacer regions and intervening 5.8S nrRNA gene; LSU: partial nuclear ribosomal DNA large subunit.

Table S2. List of taxa used for the phylogenetic analysis of *Emericellopsis* spp. and their GenBank accession number.

		GenBank accession number	
**Species**	**Strain**	**ITS**	**TUB**
Acremonium exuviarum	UAMH 9995	AY882946	AY882947
Acremonium fuci	CBS 112868	AY632653	AY632690
Acremonium potronii	CBS 379.70F	AY632655	AY632691
Acremonium sclerotigenum	A130	KC987166	KC987128
Emericellopsis alkalina	CBS 127350	KC987171	KC987133
Emericellopsis alkalina	CBS 120044	KC987169	KC987131
Emericellopsis alkalina	CBS 120049	KC987170	KC987132
Emericellopsis donezkii	CBS 489.71	AY632658	AY632674
Emericellopsis glabra	A.R. 3614	HM484860	HM484879
Emericellopsis humicola	CBS 180.56	AY632659	AY632675
Emericellopsis maritima	CBS 491.71	KC987175	KC987137
Emericellopsis microspora	CBS 380.62	AY632663	AY632679
Emericellopsis minima	CBS 190.55	KC987173	KC987135
Emericellopsis minima	CBS 871.68	KC987174	KC987136
Emericellopsis pallida	CBS 490.71	KC987176	KC987138
Emericellopsis pallida	CBS 624.73	AY632667	AY632683
Emericellopsis robusta	CBS 489.73	AY632664	AY632680
Emericellopsis salmosynnemata	CBS 382.62	AY632666	AY632682
Emericellopsis stolkiae	CBS 159.71	AY632668	AY632684
Emericellopsis synnematicola	CBS 176.60	AY632665	AY632681
Emericellopsis terricola	CBS 229.59	AY632662	AY632678

Table S3. List of fungal strains isolated from *G. compressa* with Mycotheca Universitatis Taurinensis (MUT) and GenBank accession number.

MUT CODE	Taxa	GenBank accession number			
		ITS	LSU	ACT	TUB
2282	*Coprinellus* sp.	MF140467	MF140459		
2332		MF140469	MF140461		
2288	*Ceriporia lacerata*	MF125292	MF125289		
2307	*Cladosporium allicinum*			-	
2313				MH383514	
2314	*Cladosporium cladosporioides*			MH383515	
2315	*Cladosporium pseudocladosporioides*			MH383516	
2273	*Emericellopsis pallida*	MH399734	MG845233		MH480660
2274	*Emericellopsis pallida*	MH399735	MG845234		MH480663
2316	*Eurotium chevalieri*	MH399736			MH383517
2334	*Euthypella scoparia*	MH399739	MG845235		
2317	*Fusarium solani*	MH399737			
2321	*Penicillium chrysogenum*				MH383518
2328	*Penicillium oxalicum*	MH399738			
2322	*Penicillium paneum*				MH383519
2326					MH383520
2331	*Psathyrella candolleana*	MF125293	MF125290		
2415	*Rhodotorula mucilaginosa*	MF423718			
-	*Tetracladium* sp.				
2410	*Tolypocladium cylindrosporum*	MH399740			
2413		MH399741			

Echinulin (1):

^{1}H NMR (500 MHz, $CDCl_3$, *δ*/ppm, *J*/Hz): 8.05 (1H, s, 1-NH), 7.13 (1H, s, H-4), 6.80 (1H, s, H-6), 6.10 (1H, dd, *J* = 17.3, 10.6, H-2′), 5.42 (1H, t, *J* = 6.8, H-2′′′), 5.35 (1H, m, H-2″), 5.14 (2H, m, H-3′), 4.41 (1H, m, H-9), 4.10 (1H, q, *J* = 6.8, H-12), 3.66 (1H, dd, *J* = 14.6, 3.2, H-8), 3.53 (2H, d, *J* = 7.1, H-1′′′), 3.39 (2H, d, *J* = 7.1, H-1″), 3.18 (1H, dd, *J* = 14.6, 11.8, H-8), 1.87 (3H, s, CH_3-3′′′), 1.81 (3H, s, CH_3-3′′′), 1.74 (3H, overlapped, CH_3-3″), 1.51 (3H, overlapped, CH_3-1′).

^{13}C NMR (125 MHz, $CDCl_3$, *δ*/ppm): 168.6 (C-10), 167.9 (C-13), 145.9 (C-2′), 141.5 (C-2), 134.0 (C-5), 133.1 (C-3′′′), 132.4 (C-7a), 131.7 (C-3″), 129.1 (C-3a), 124.6 (C-2″), 123.5 (C-7), 123.0 (C-2′′′), 123.0 (C-6), 115.2 (C-4), 112.5 (C-3′), 104.2 (C-3), 54.7 (C-9), 51.0 (C-12), 39.1 (C-1′), 34.7 (C-1″), 31.5 (C-1′′′), 29.6 (C-8), 28.1 (CH_3-1′), 27.9 (CH_3-1′), 25.9 (CH_3-3′′′), 25.9 (CH_3-3″), 20.0 (C-15), 18.1 (CH_3-3′′′), 18.1(CH_3-3″).

LR-ESI-MS: *m*/*z* 462.2 $[M + H]^+$.

Neoechinulin A (2):

^{1}H NMR (500 MHz, $CDCl_3$, *δ*/ppm, *J*/Hz): 8.33 (1H, s, 1-NH), 7.36 (1H, d, *J* = 8.0, H-4), 7.27 (1H, d, *J* = 7.5, H-7), 7.18 (1H, m, H-6), 7.07 (1H, m, H-5), 6.08 (1H, dd, *J* = 17.4, 10.6, H-2′), 5.21 (2H, m, H-3′), 4.22 (1H, q, *J* = 6.0, H-12), 1.60 (6H, overlapped, CH_3-1′), 1.53 (3H, d, *J* = 6.0, CH_3-12).

^{13}C NMR (125 MHz, $CDCl_3$, *δ*/ppm): 165.8 (C-13), 160.0 (C-10), 144.4 (C-2′), 143.9 (C-2), 134.4 (C-7a), 126.2 (C-3a), 124.5 (C-9), 122.5 (C-6), 121.2 (C-5), 119.0 (C-4), 113.5 (C-8), 112.1 (C-7), 111.4 (C-3′), 103.1 (C-3), 51.8 (C-12), 39.4 (C-1′), 27.5 (CH_3-1′), 21.0 (CH_3-12).

LR-ESI-MS: *m*/*z* 324.1 $[M + H]^+$.

Physcion (3):

^{1}H NMR (500 MHz, $CDCl_3$, *δ*/ppm, *J*/Hz): 12.31 (1H, s, 5-OH), 12.11 (1H, s, 4-OH), 7.63 (1H, s, H-1), 7.37 (1H, d, *J* = 2.0, H-8), 7.08 (1H, s, H-3), 6.69 (1H, d, *J* = 2.0, H-6), 3.94 (3H, s, 7-OCH_3), 2.45 (3H, s, 2-CH_3).

^{13}C NMR (125 MHz, $CDCl_3$, δ/ppm): 190.9 (C-10), 182.2 (C-9), 166.7 (C-7), 165.3 (C-5), 162.6 (C-4), 148.6 (C-2), 135.4 (C-8a), 133.4 (C-1a), 124.7 (C-3), 121.4 (C-1), 113.8 (C-4a), 110.4 (C-5a), 108.4 (C-6), 106.9 (C-8), 56.2 (3-OCH_3), 22.3 (2-CH_3).

LR-ESI-MS: *m/z* 285.1 $[M + H]^+$.

Dihydroauroglaucin (4):

^1H NMR (500 MHz, $CDCl_3$, δ/ppm, *J*/Hz): 11.8 (1H, s, 5-OH), 10.01 (1H, s, 1-CHO), 6.90 (1H, s, H-4), 6.56 (1H, d, *J* = 15.9, H-1′), 6.44 (1H, ddd, *J* = 15.9, 10.2, 0.6, H-2′), 6.27 (1H, ddt, *J* = 15.1, 10.2, 1.4, H-3′), 5.89 (dt, *J* = 15.0, 7.0 Hz, H-4′), 5.30 (1H, m, H-2′′), 4.93 (1H, 2-OH), 3.31 (2H, d, *J* = 7.5, H-1′′), 2.15 (2H, m, H-5′), 1.76 (3H, s, H-4′′), 1.70 (3H, s, H-5′′), 1.47 (2H, m, H-6′), 0.94 (3H, t, *J* = 6.0, H-7′).

^{13}C NMR (125 MHz, $CDCl_3$, δ/ppm): 196.4 (1-CHO), 155.4 (C-5), 145.2 (C-2), 140.1 (C-4′), 139.1 (C-2′), 134.2 (C-3′′), 130.6 (C-3), 129.7 (C-6), 125.3 (C-4), 124.2 (C-2′′), 121.1 (C-3′), 119.5 (C-1′), 117.2 (C-1), 35.0 (C-5′), 27.4 (C-1′′), 26.0 (C-4′′), 22.4 (C-6′), 17.9 (C-5′′), 13.9 (C-7′).

LR-ESI-MS: *m/z* 301.1 $[M + H]^+$.

Flavoglaucin (5):

^1H NMR (500 MHz, $CDCl_3$, δ/ppm, *J*/Hz): 11.91 (1H, s, 5-OH), 10.25 (1H, s, 1-CHO), 6.89 (1H, s, H-4), 5.28 (1H, m, H-2″), 4.43 (1H, 2-OH), 3.29 (2H, d, *J* = 7.5, H-1″), 2.88 (2H, t, *J* = 7.5, H-1′), 1.76 (3H, s, H-4″), 1.69 (3H, s, H-5″), 1.57 (2H, m, H-2′), 1.40 (2H, m, H-3′), 1.27 (6H, overlapped, H-4′, 5′, 6′), 0.88 (3H, t, *J* = 6.0, H-7′).

^{13}C NMR (125 MHz, $CDCl_3$, δ/ppm): 195.7 (1-CHO), 155.9 (C-5), 145.1 (C-2), 134.0 (C-3′′), 128.7 (C-3, C-6), 125.8 (C-4), 121.3 (C-2′′), 117.5 (C-1), 32.2 (C-5′), 31.9 (C-4′), 29.7 (C-3′), 29.3 (C-2′), 27.1 (C-1′′), 25.9 (C-4′′), 24.1 (C-1′), 22.8 (C-6′), 17.9 (C-5′), 14.2 (C-7′).

LR-ESI-MS: *m/z* 305.1 $[M + H]^+$.

Isodihydroauroglaucin (6):

^1H NMR (500 MHz, $CDCl_3$, δ/ppm, *J*/Hz): 11.93 (1H, s, 5-OH), 10.23 (1H, s, 1-CHO), 6.90 (1H, s, H-4), 6.01 (1H, overlapped, H-4′), 5.99 (1H, overlapped, H-5′), 5.59 (1H, overlapped, H-3′), 5.59 (1H, overlapped, H-6′), 5.30 (1H, m, H-2″), 4.43 (1H, 2-OH), 3.29 (2H, d, *J* = 7.5, H-1″), 2.98 (2H, t, *J* = 7.5, H-1′), 2.34 (2H, m, H-2′), 1.76 (3H, s, H-4″), 1.73 (3H, d, *J* = 6.0, H-7′), 1.69 (3H, s, H-5″).

^{13}C NMR (125 MHz, $CDCl_3$, δ/ppm): 195.5 (1-CHO), 156.0 (C-5), 145.2 (C-2), 134.1 (C-3″), 132.2 (C-5′), 131.3 (C-6′), 129.5 (C-4′), 129.1 (C-6), 128.5 (C-3′), 127.5 (C-3), 126.0 (C-4), 117.4 (C-1), 34.4 (C-2′), 29.9 (C-2′′), 27.2 (C-1′′), 26.0 (C-4′′), 24.3 (C-1′), 18.2 (C-5′′), 17.9 (C-7′).

LR-ESI-MS: *m/z* 301.1 $[M + H]^+$.

Neoechinulin D (7):

^1H NMR (500 MHz, $CDCl_3$, δ/ppm, *J*/Hz): 8.18 (1H, s, 1-NH), 7.16 (1H, m, H-4), 7.21 (1H, s, H-7), 7.00 (1H, dd, *J* = 8.0, 1.5, H-5), 6.05 (1H, dd, *J* = 17.4, 10.6, H-2′), 5.23 (1H, m, H-2″), 5.21 (2H, m, H-3′), 4.29 (1H, q, *J* = 6.0, H-12), 3.43 (2H, d, *J* = 7.5, H-1″), 1.76 (3H, s, H-4″), 1.74 (3H, s, H-5″), 1.60 (6H, overlapped, CH_3-1′), 1.51 (3H, d, *J* = 6.0, CH_3-12).

^{13}C NMR (125 MHz, $CDCl_3$, δ/ppm): 165.7 (C-13), 160.0 (C-10), 144.5 (C-2′), 143.4 (C-2), 124.3 (C-6), 123.8 (C-7a), 122.4 (C-3″), 124.3 (C-3a), 123.8 (C-9), 122.4 (C-5), 118.9 (C-4), 113.4 (C-8), 112.4 (C-7), 110.6 (C-3′), 103.2 (C-3), 51.8 (C-12), 39.4 (C-1′), 27.6 (CH_3-1′), 27.5 (CH_3-1′), 25.9 (CH_3-4″), 21.1 (CH_3-12), 18.0 (CH_3-5″).

LR-ESI-MS: *m/z* 392.2 $[M + H]^+$.

Asperflavin (8):

^1H NMR (500 MHz, CD_3OD, δ/ppm, *J*/Hz): 6.81 (1H, s, H-10), 6.54 (1H, d, *J* = 2.0, H-5), 6.45 (1H, d, *J* = 2.0, H-7), 3.92 (3H, s, CH_3O-8), 3.01 (2H, s, H-4), 2.79 (1H, d, *J* = 16.9, H-2a), 2.79 (1H, d, *J* = 16.9, H-2b), 1.37 (3H, s, CH_3-3).

^{13}C NMR (125 MHz, CD_3OD, δ/ppm,): 204.5 (C-1), 166.9 (C-9), 163.3 (C-8), 162.4 (C-6), 144.1 (C-10a), 138.8 (C-4a), 118.1 (C-10), 110.7 (C-8a), 103.8(C-9a), 99.3 (C-5), 97.8 (C-7), 71.7 (C-3), 56.7 (CH_3O-8), 52.9 (C-2), 44.6 (C-4), 29.3 (CH_3-3).

LR-ESI-MS: *m/z* 287.1 $[M - H]^-$.

Cinnalutein (9):

^1H NMR (500 MHz, CD_3OD, δ/ppm, *J*/Hz): 7.51 (1H, s, H-4), 7.22 (1H, s, H-5), 6.94 (1H, s, H-7), 3.94 (3H, s, 6-OCH_3), 2.43 (3H, s, 3-$COCH_3$).

^{13}C NMR (125 MHz, CD_3OD, δ/ppm): 188.3 (C-9), 184.1 (C-10), 174.3 (-COOH), 170.2 (C-1 or C-8), 165.4 (C-6), 159.2 (C-1 or C-8), 142.0 (C-2 or C3), 139.5 (C-10a), 138.8 (C-4a), 131.9 (C-2 or C-3), 121.1 (C-4), 116.2 (C-9a), 109.5 (C-8a), 105.9 (C-5), 102.9 (C-7), 56.7 (1C, 6-OCH_3), 20.2 (1C, 3-$COCH_3$).

LR-ESI-MS: *m/z* 327.1 $[M + H]^-$.

Cyclo-L-Trp- L-Ala (10):

^1H NMR (500 MHz, $CDCl_3$, δ/ppm, *J*/Hz): 8.52 (1H, s, 1-NH), 7.60 (1H, d, *J* = 8.0, H-4), 7.32 (1H, d, *J* = 8, H-7), 7.06 (2H, m, H-2, H-6), 6.99 (1H, t, *J* = 8, H-5), 4.27 (1H, t, *J* = 4.5, H-9), 3.59 (1H, q, *J* = 7.0, H-12), 3.45 (1H, dd, *J* = 14.9, 3.9, H-8), 3.14 (1H, dd, *J* = 14.9, 3.9, H-8), 0.36 (3H, d, *J* = 7.0, H-13).

^{13}C NMR (125 MHz, $CDCl_3$, δ/ppm): 170.6 (C-10), 169.5 (C-13), 137.8 (C-7a), 129.2 (C-3a), 125.8 (C-2), 122.5 (C-5), 120.1 (C-4), 120.0 (C-6), 112.1 (C-7), 109.3 (C-3), 57.5 (C-9), 51.7 (C-12), 30.8 (C-8), 20.0 (C-15).

LR-ESI-MS: *m/z* 258.1 $[M + H]^+$.

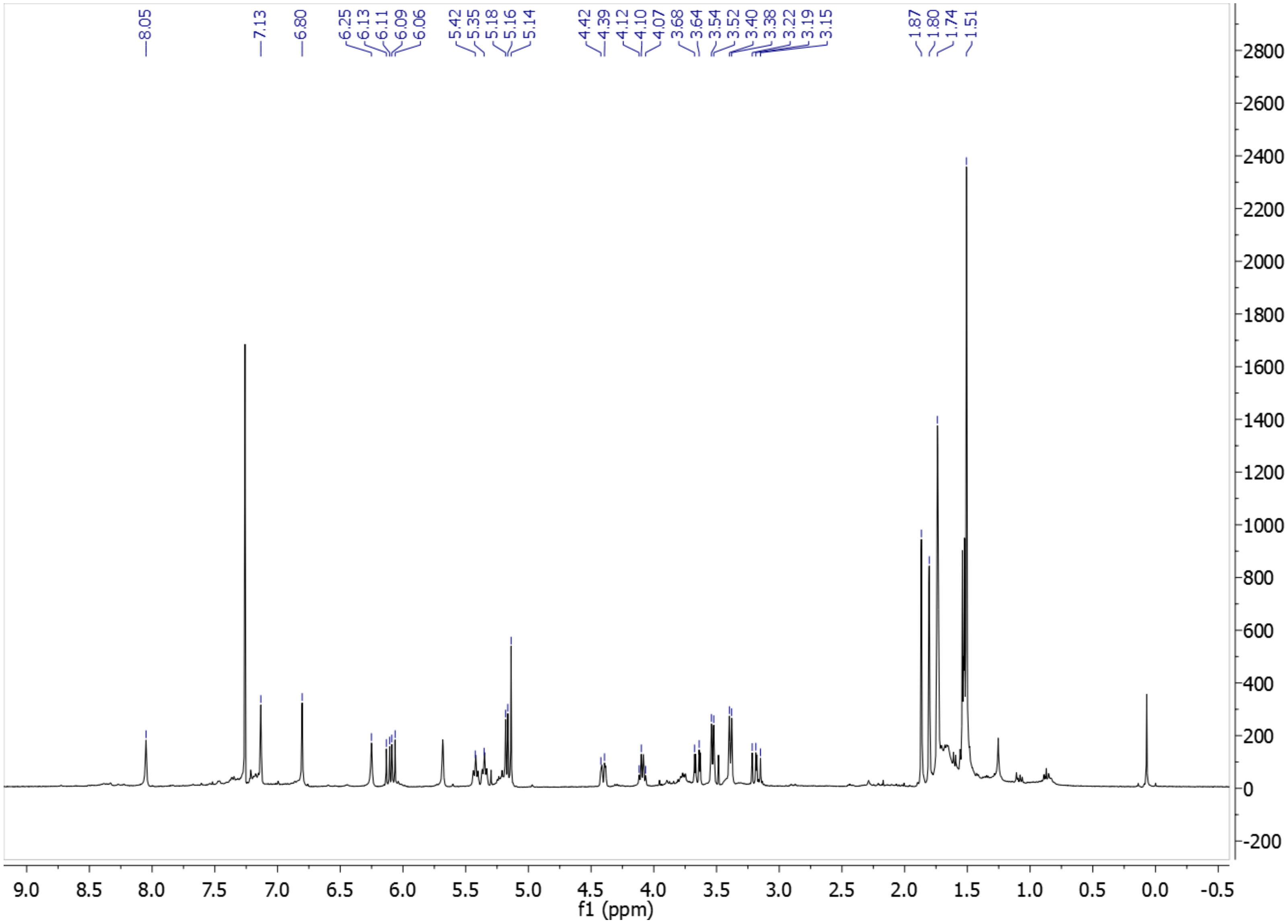

Figure S1. ^{1}H NMR spectrum of echinulin **(1)** in $CDCl_3$ at 500 MHz.

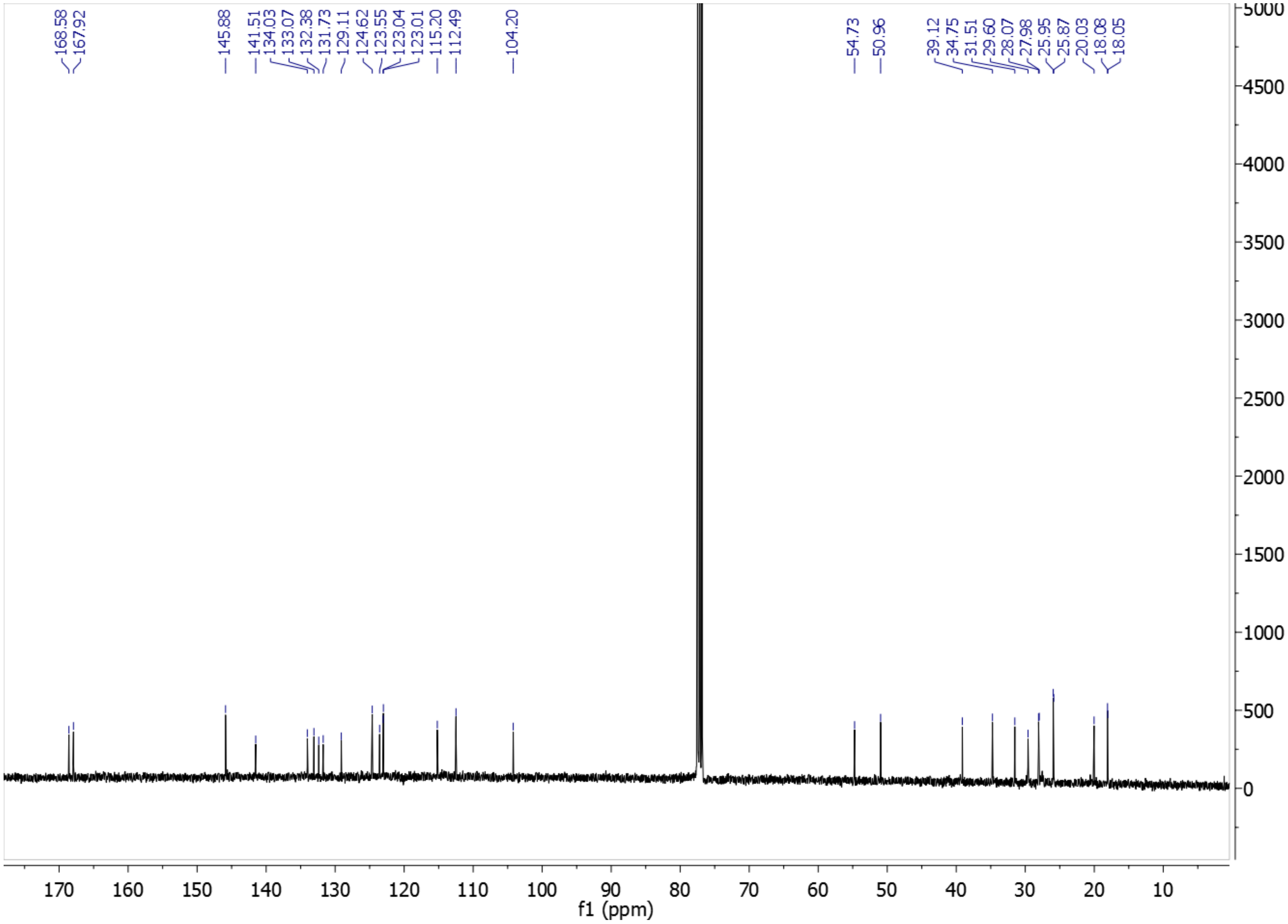

Figure S2. ^{13}C NMR spectrum of echinulin **(1)** in $CDCl_3$ at 125 MHz.

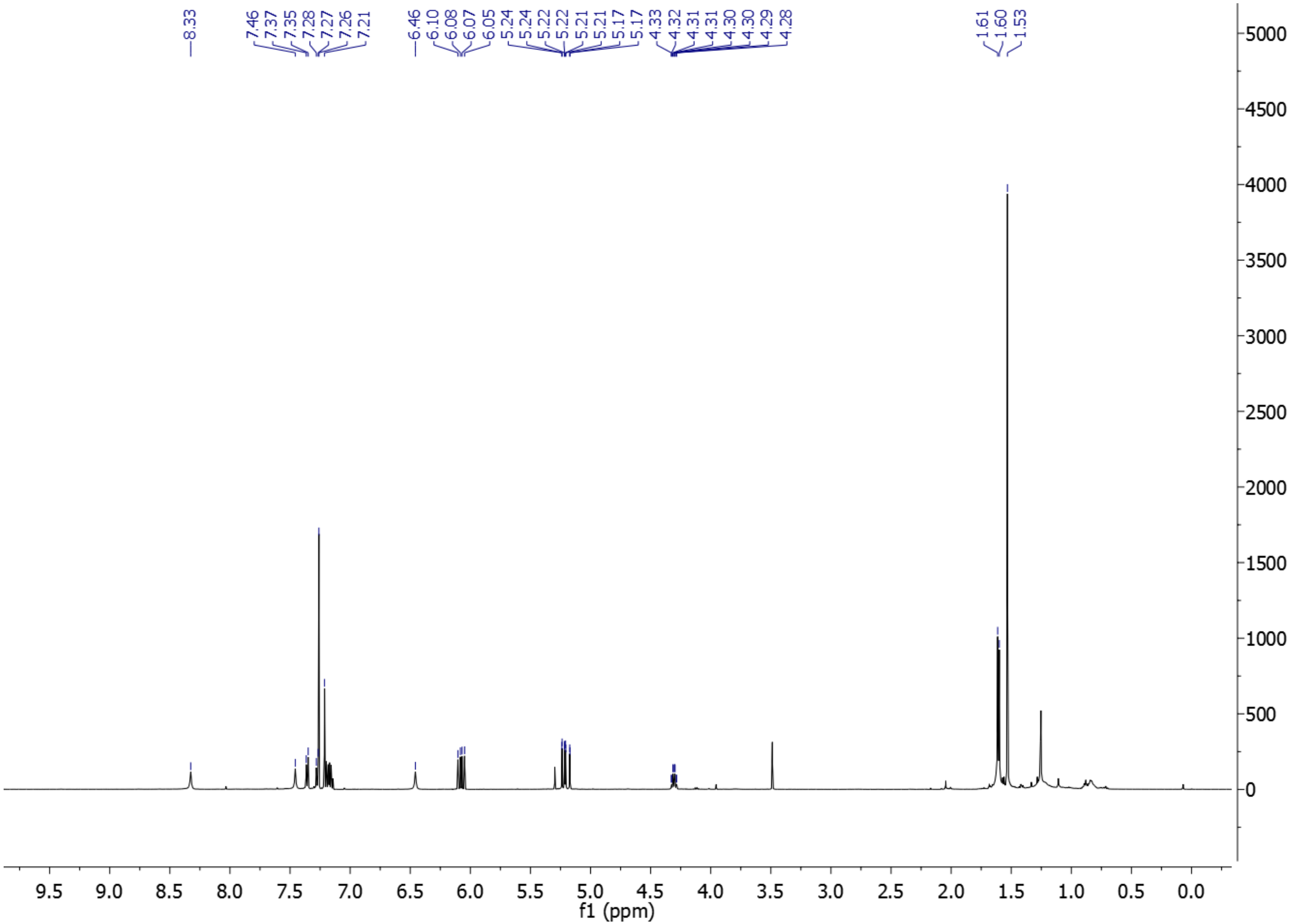

Figure S3. ^{1}H NMR spectrum of neoechinulin A **(2)** in $CDCl_3$ at 500 MHz.

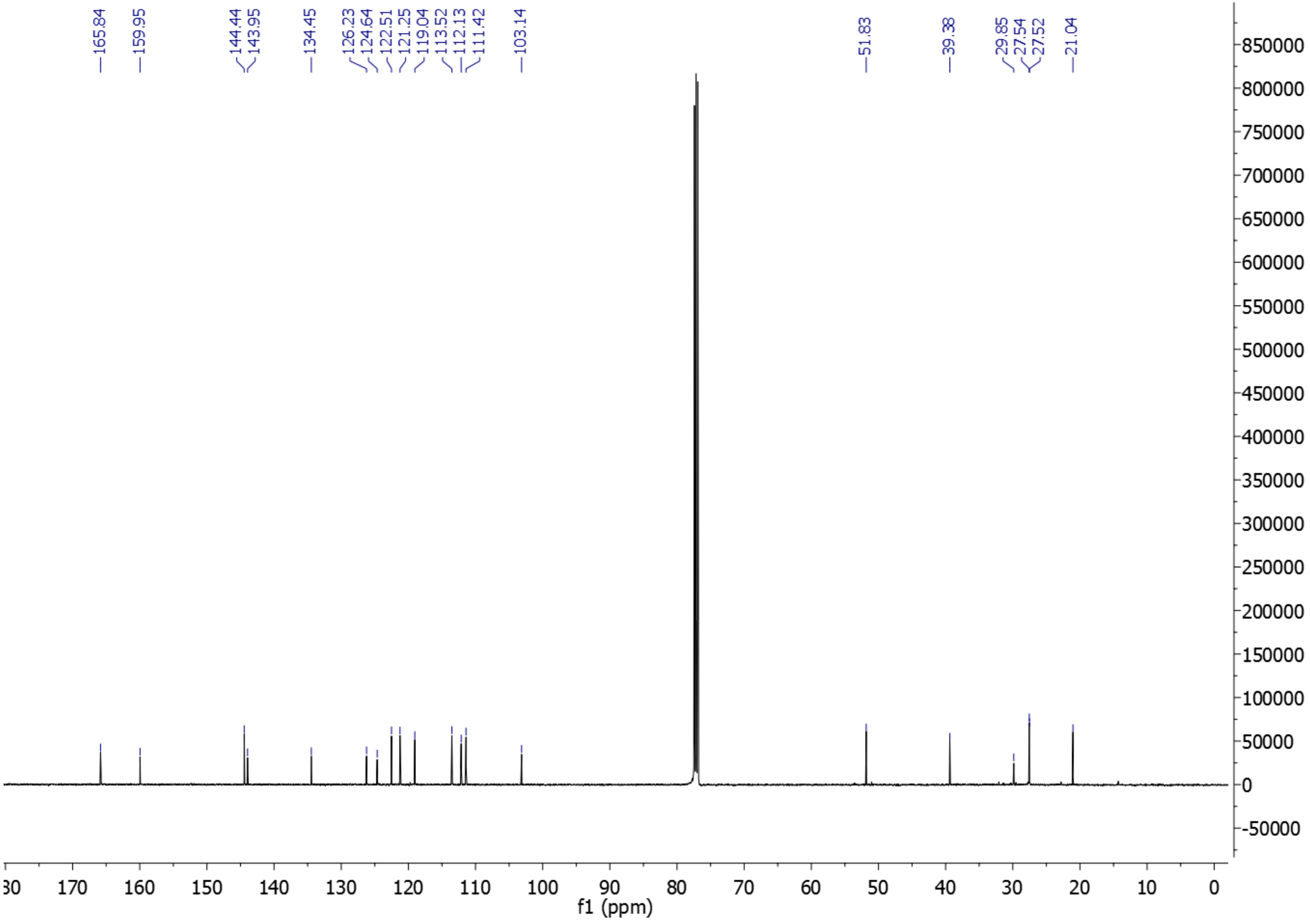

Figure S4. ^{13}C NMR spectrum of neoechinulin A **(2)** in $CDCl_3$ at 125 MHz.

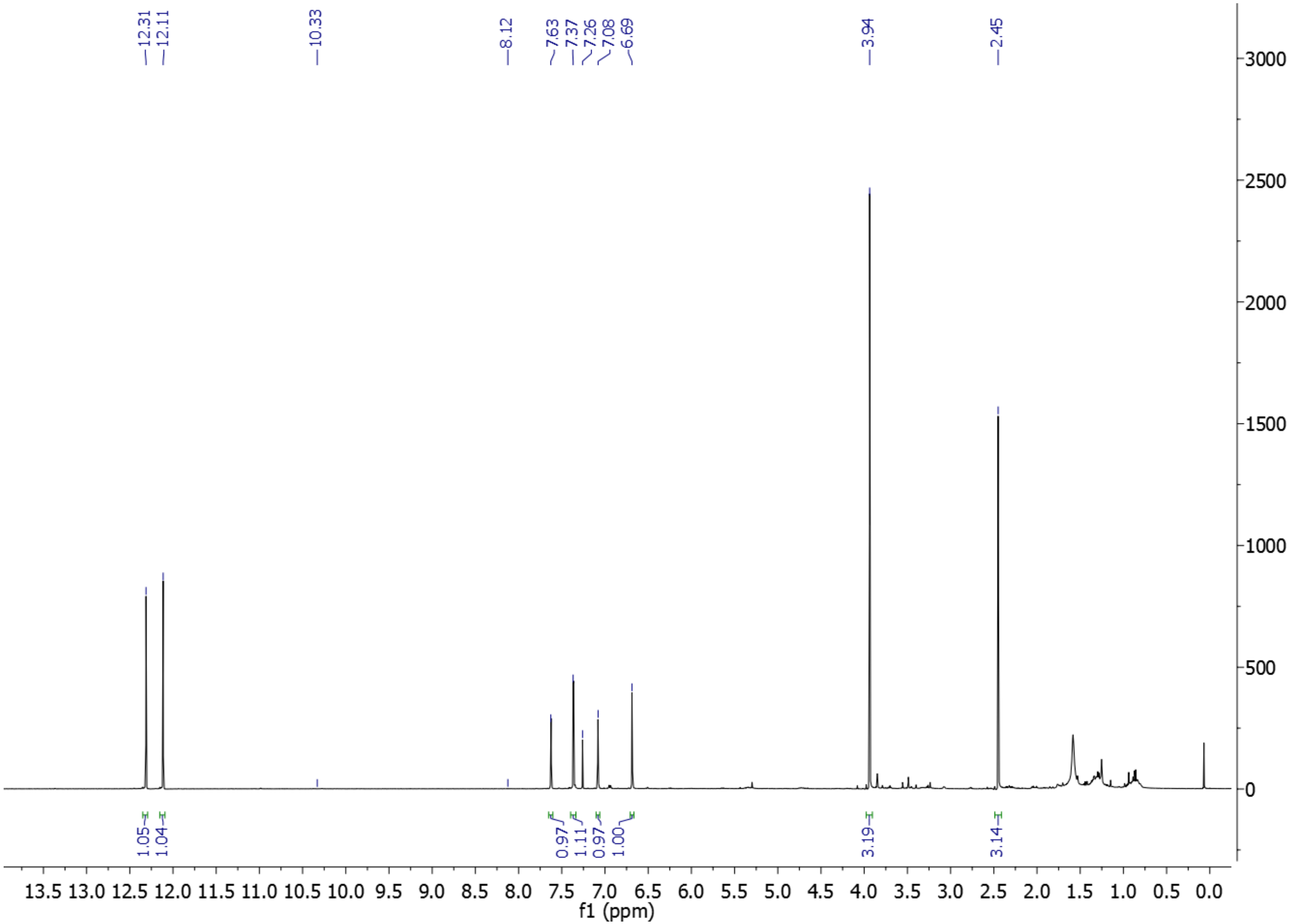

Figure S5. ^{1}H NMR spectrum of physcion **(3)** in $CDCl_3$ at 500 MHz.

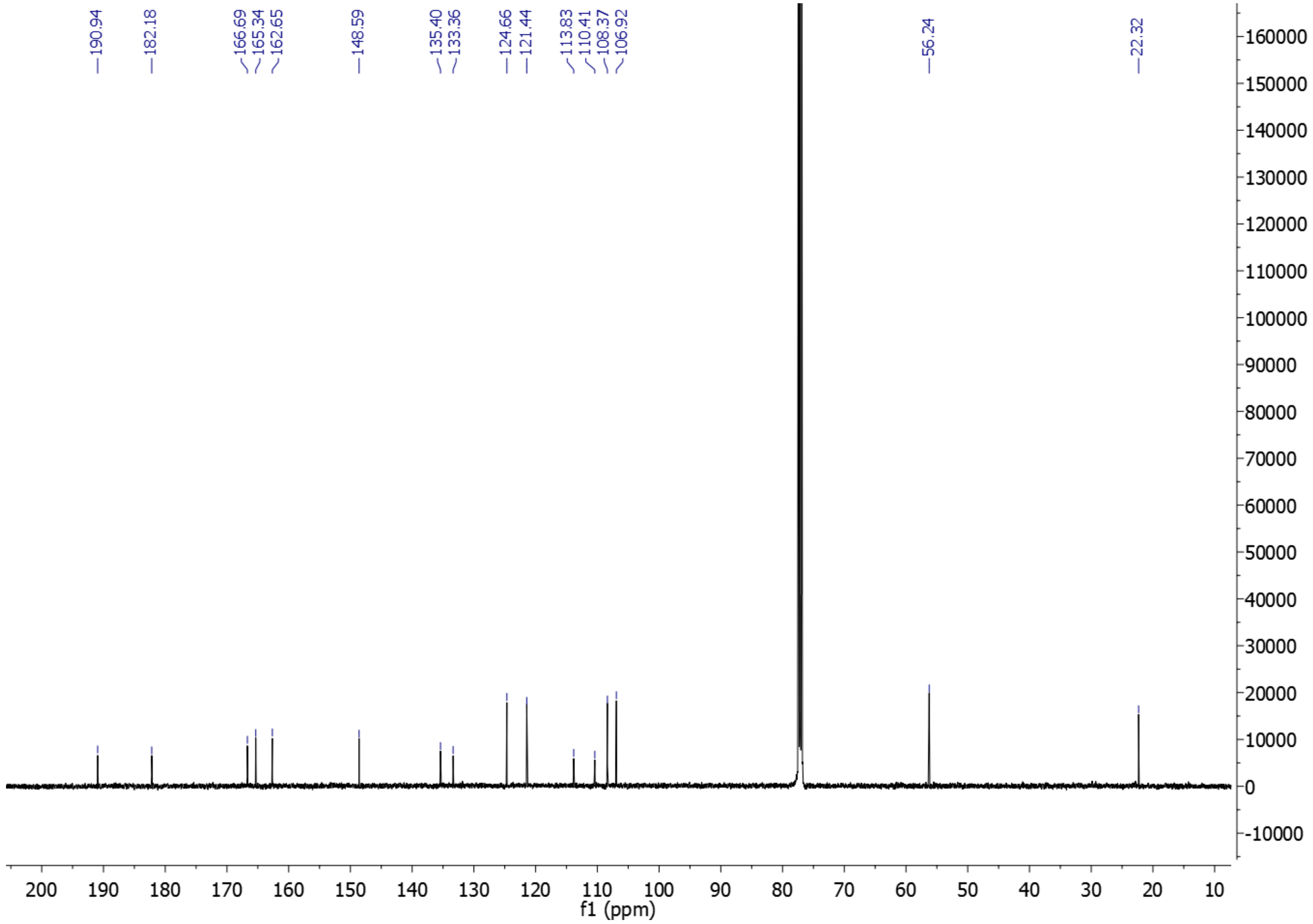

Figure S6. ^{13}C NMR spectrum of physcion **(3)** in $CDCl_3$ at 125 MHz.

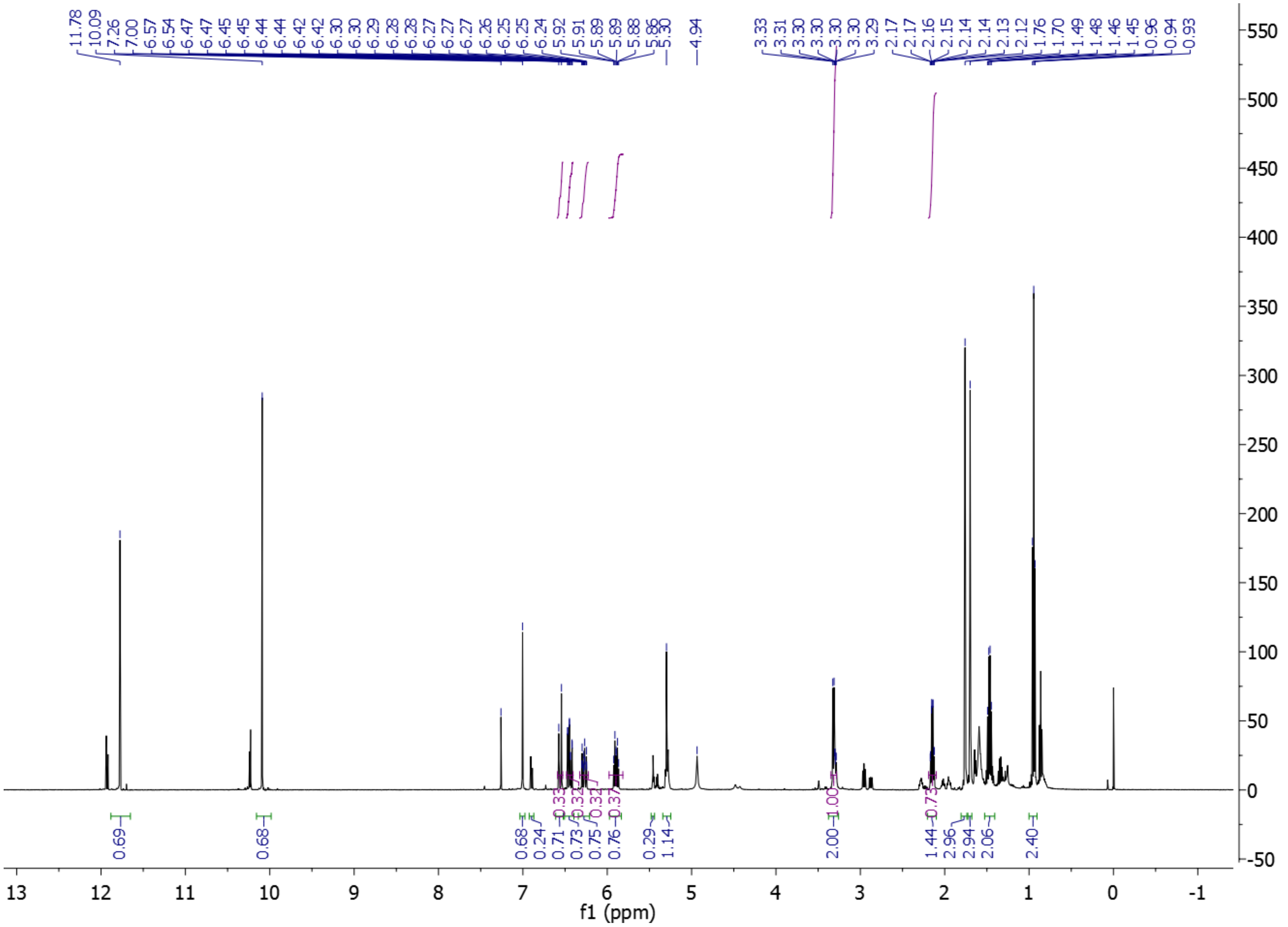

Figure S7. ^{1}H NMR spectrum of dihydroauroglaucin **(4)** in $CDCl_3$ at 500 MHz.

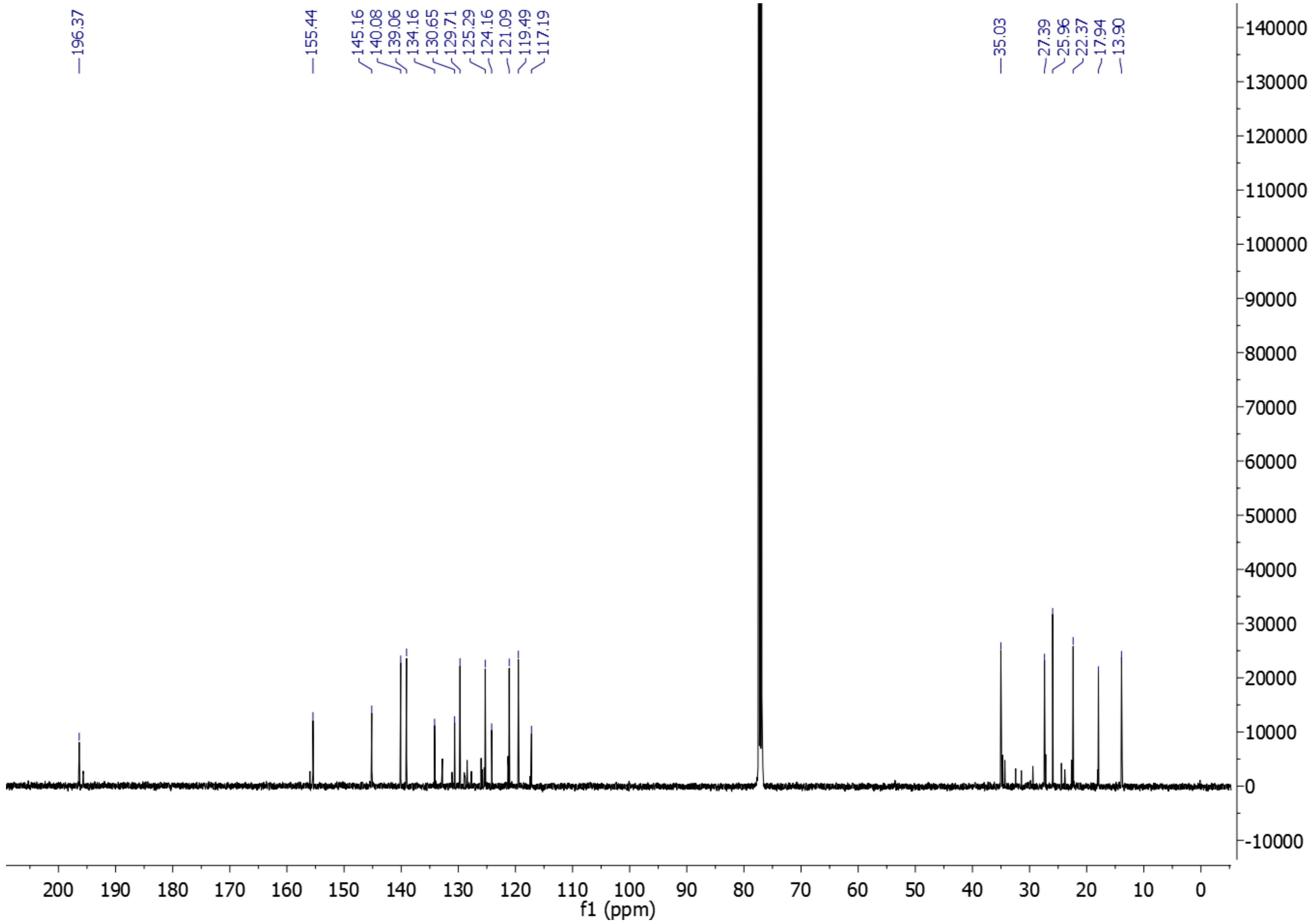

Figure S8. ^{13}C NMR spectrum of dihydroauroglaucin **(4)** in $CDCl_3$ at 125 MHz.

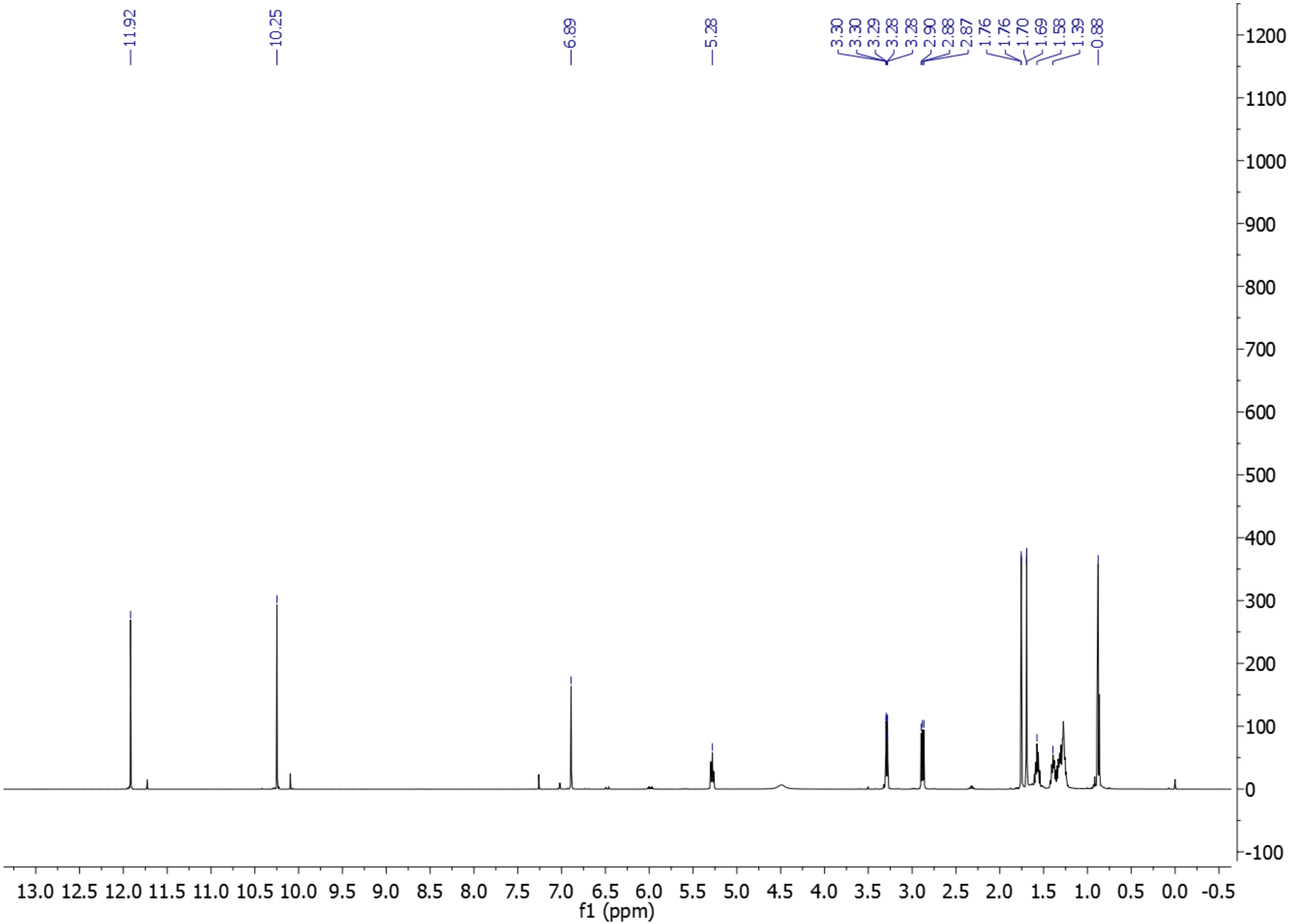

Figure S9. ^{1}H NMR spectrum of flavoglaucin **(5)** in $CDCl_3$ at 500 MHz.

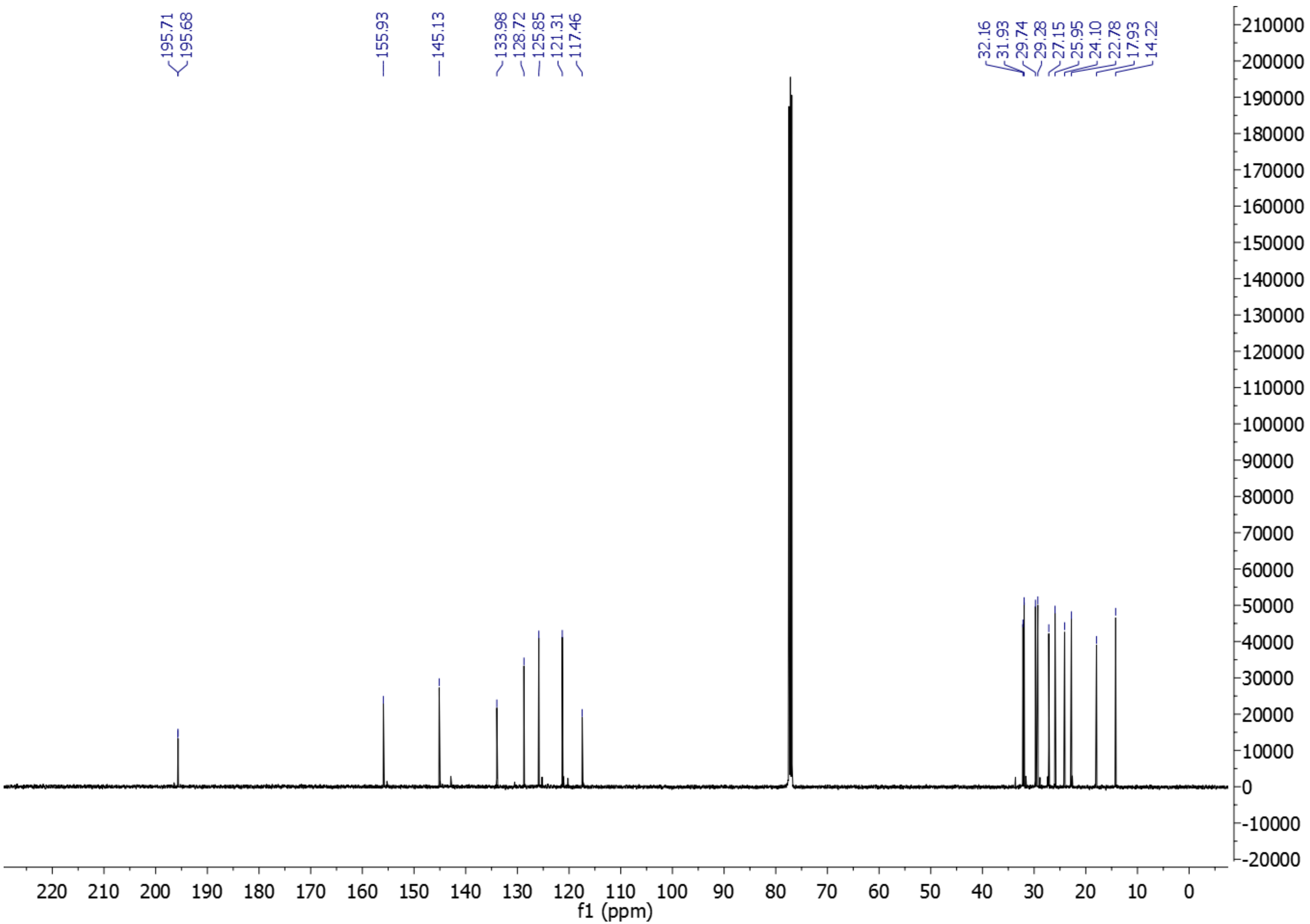

Figure S10. ^{13}C NMR spectrum of flavoglaucin **(5)** in $CDCl_3$ at 125 MHz.

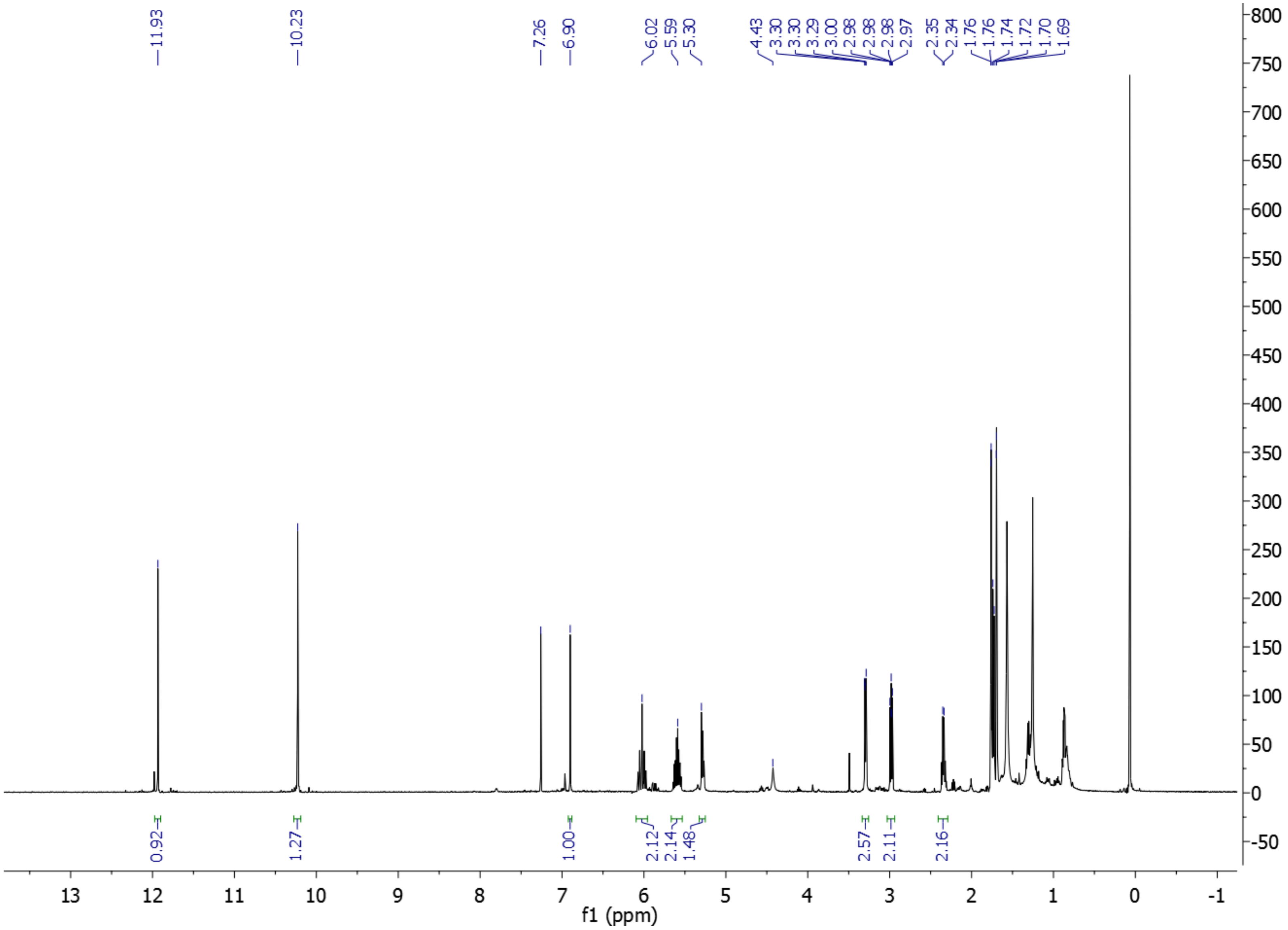

Figure S11. ^{1}H NMR spectrum of isodihydroauroglaucin **(6)** in $CDCl_3$ at 500 MHz.

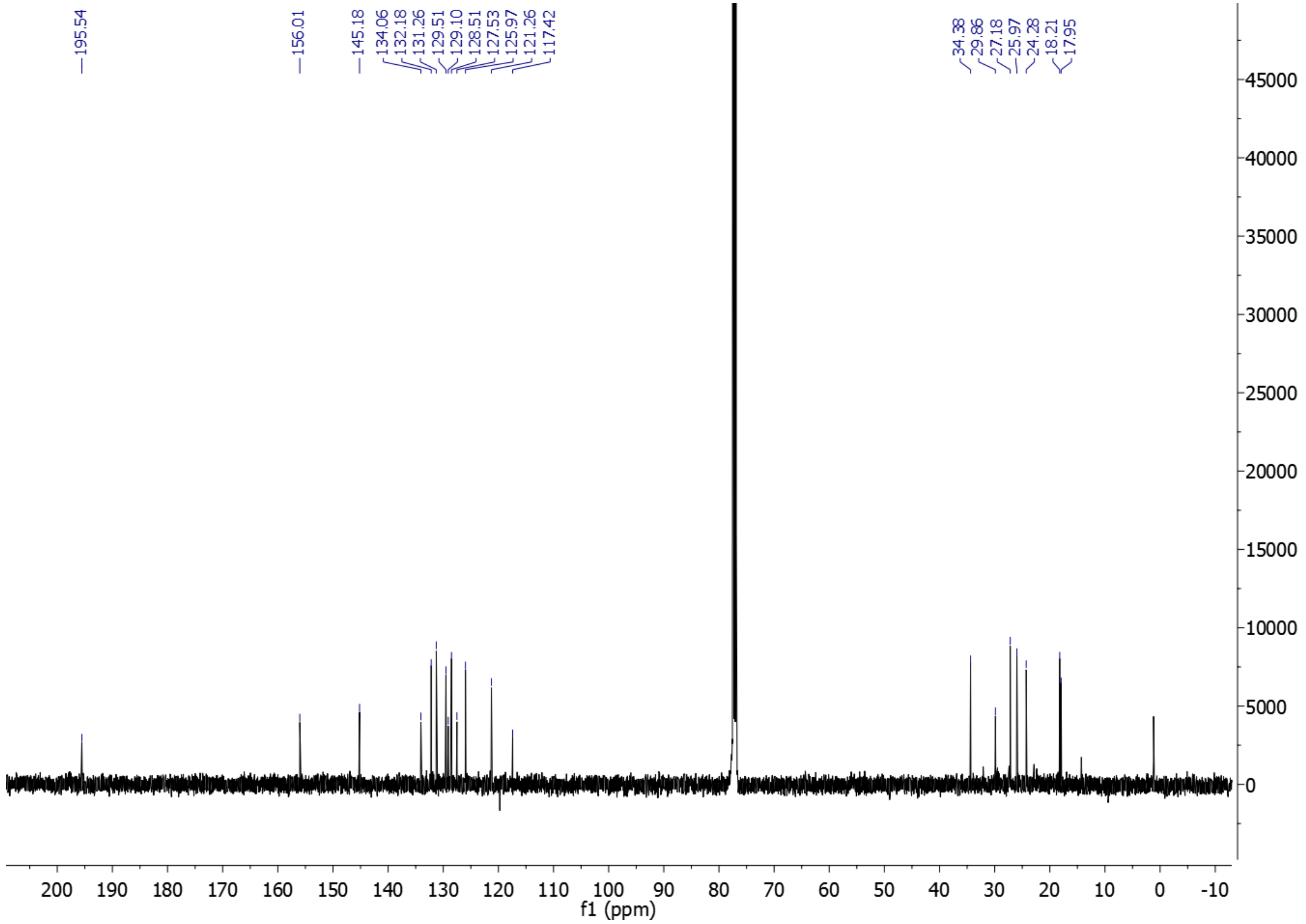

Figure S12. ^{13}C NMR spectrum of isodihydroauroglaucin **(6)** in $CDCl_3$ at 125 MHz.

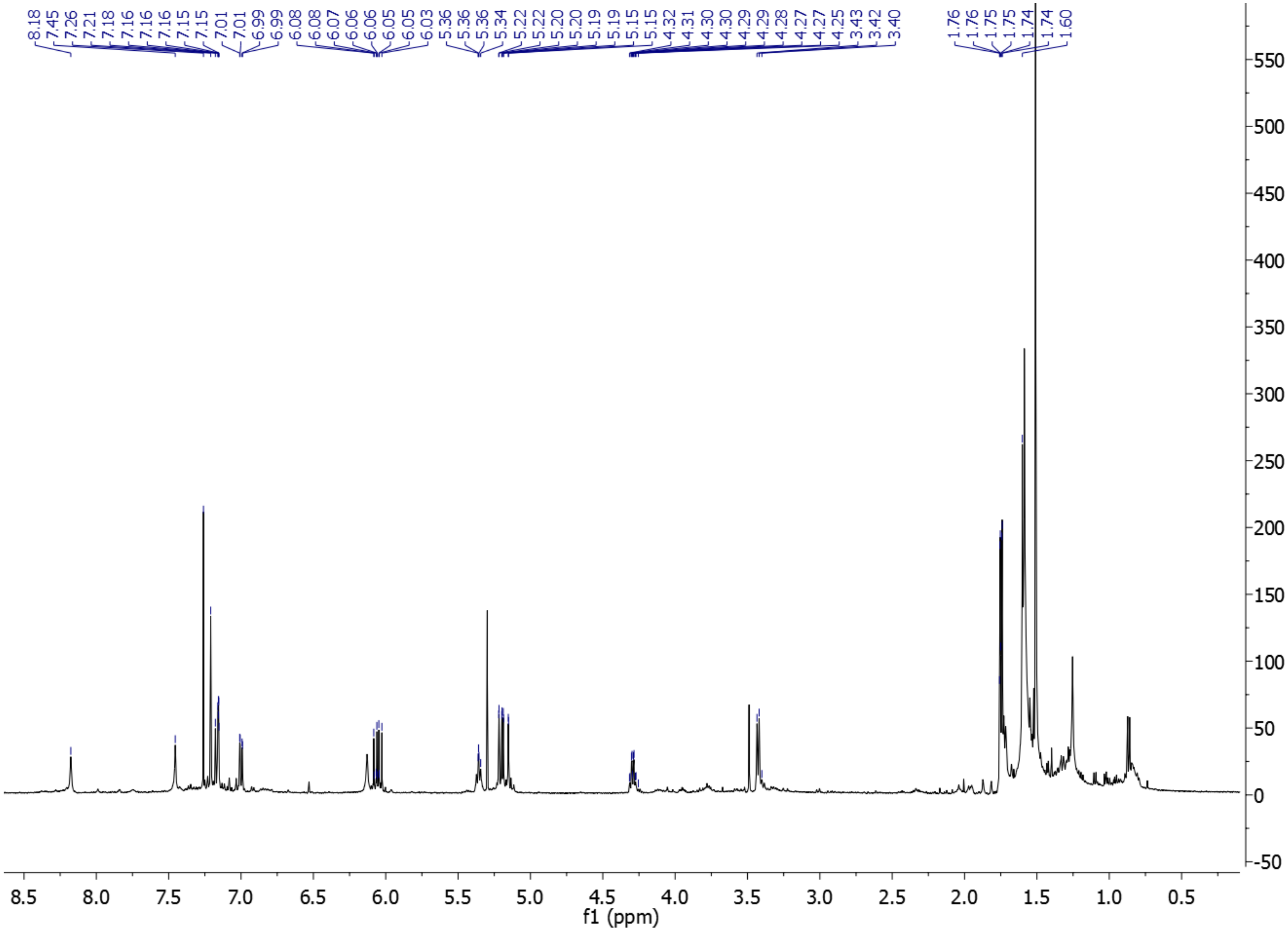

Figure S13. ^{1}H NMR spectrum of neoechinulin D **(7)** in $CDCl_3$ at 500 MHz.

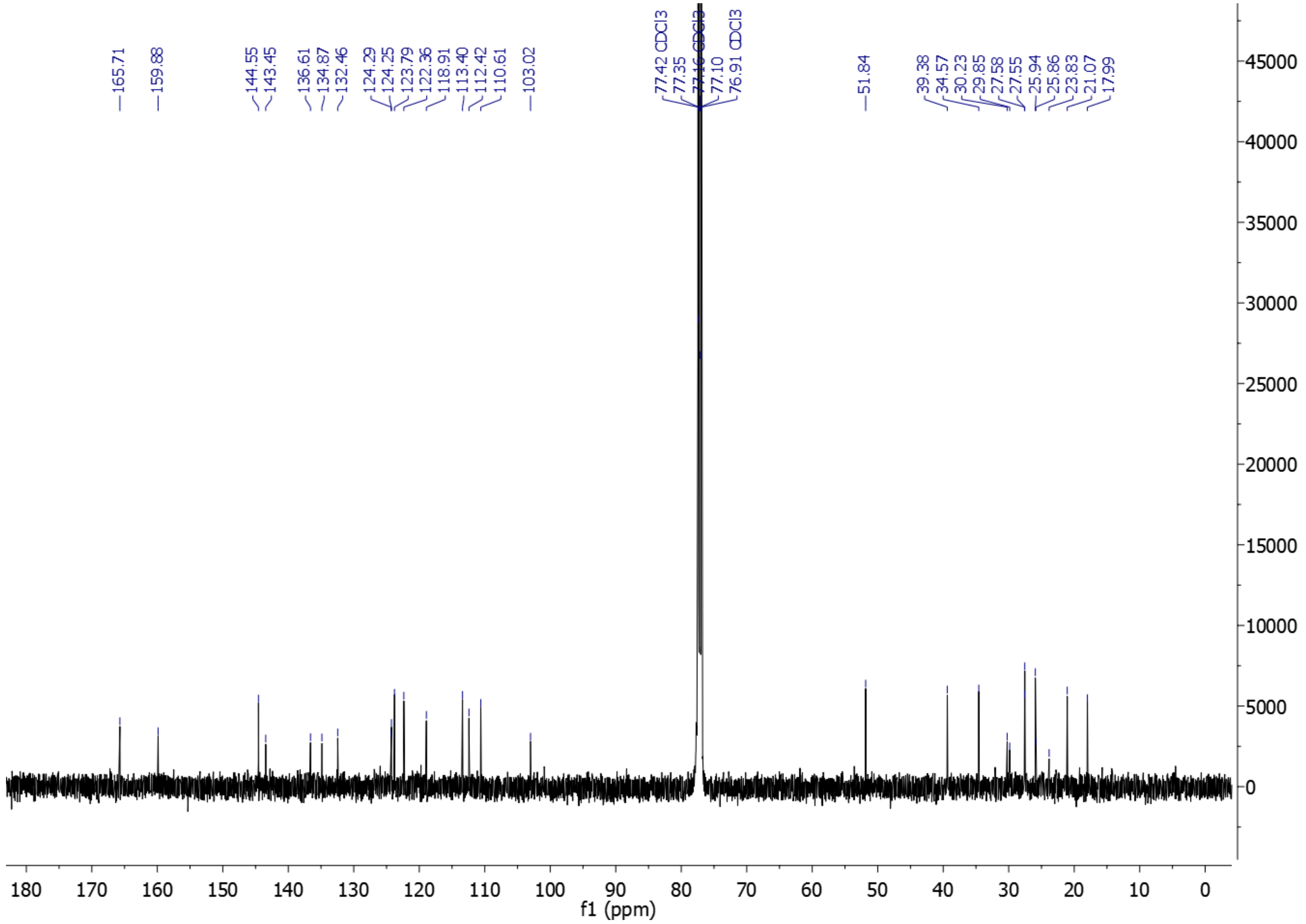

Figure S14. ^{13}C NMR spectrum of neoechinulin D **(7)** in $CDCl_3$ at 125 MHz.

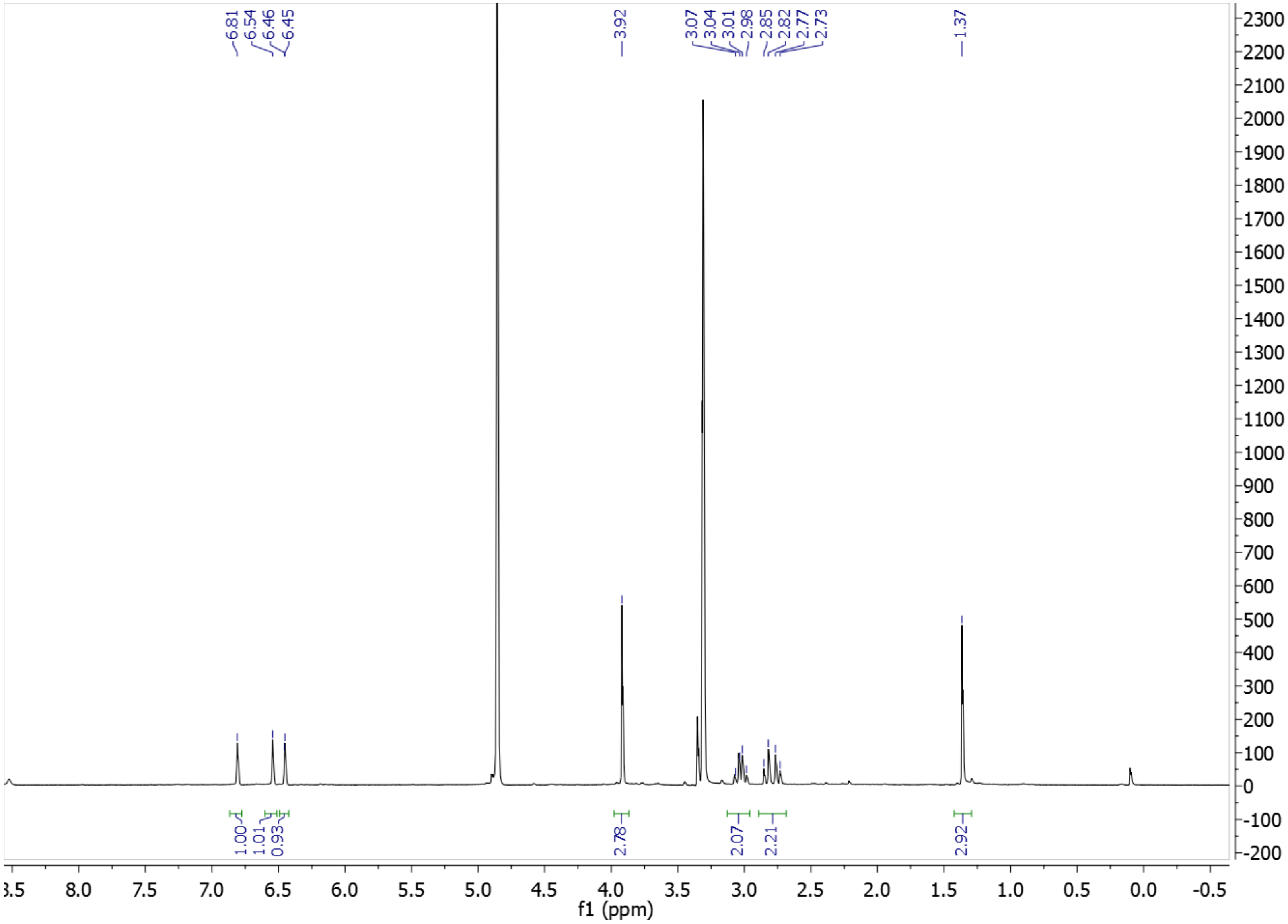

Figure S15. ^{1}H NMR spectrum of asperflavin **(8)** in CD_3OD at 500 MHz.

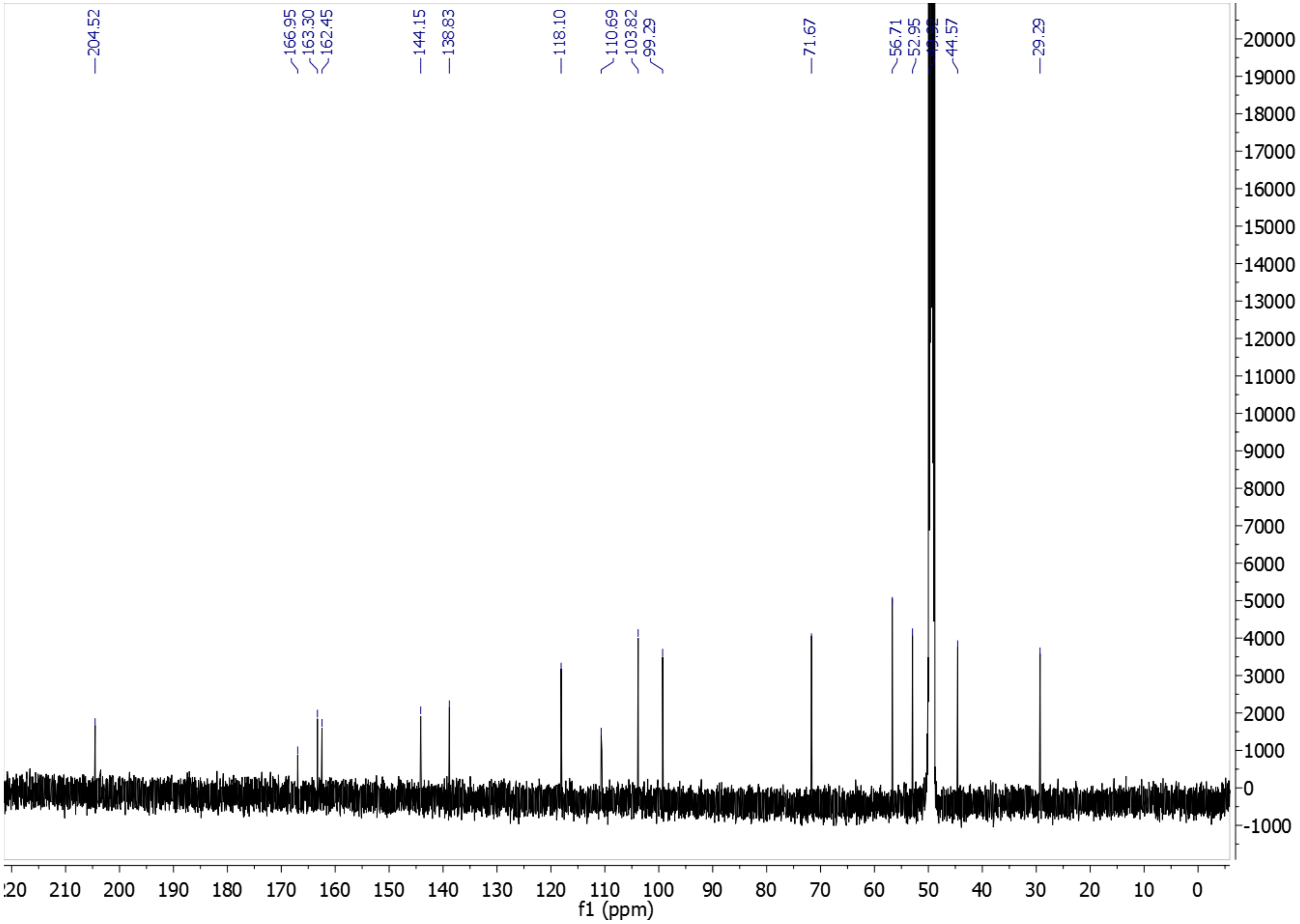

Figure S16. ^{13}C NMR spectrum of asperflavin **(8)** in CD_3OD at 125 MHz.

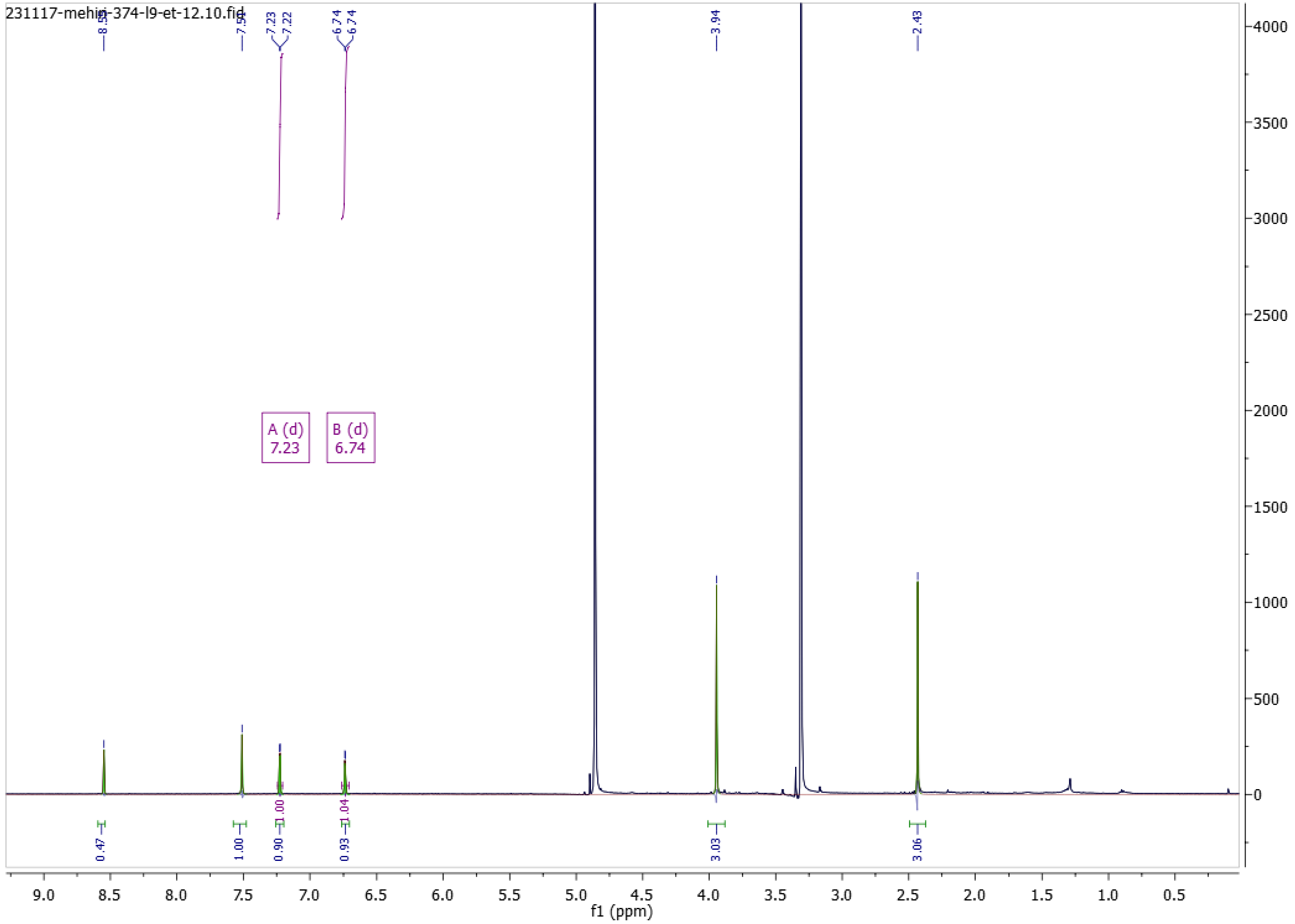

Figure S17. ^{1}H NMR spectrum of cinnalutein **(9)** in CD_3OD at 500 MHz.

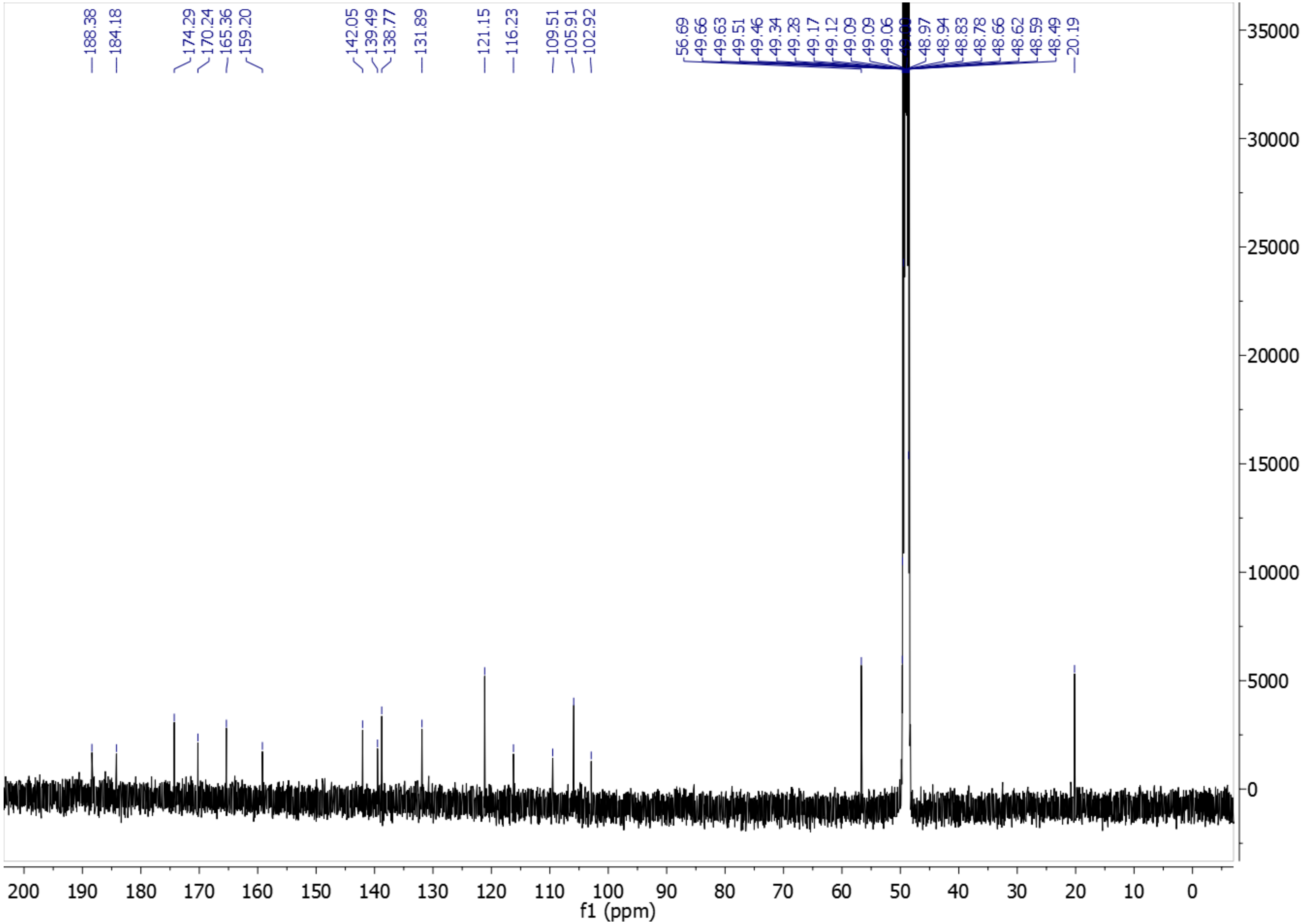

Figure S18. ^{13}C NMR spectrum of cinnalutein **(9)** in CD_3OD at 125 MHz.

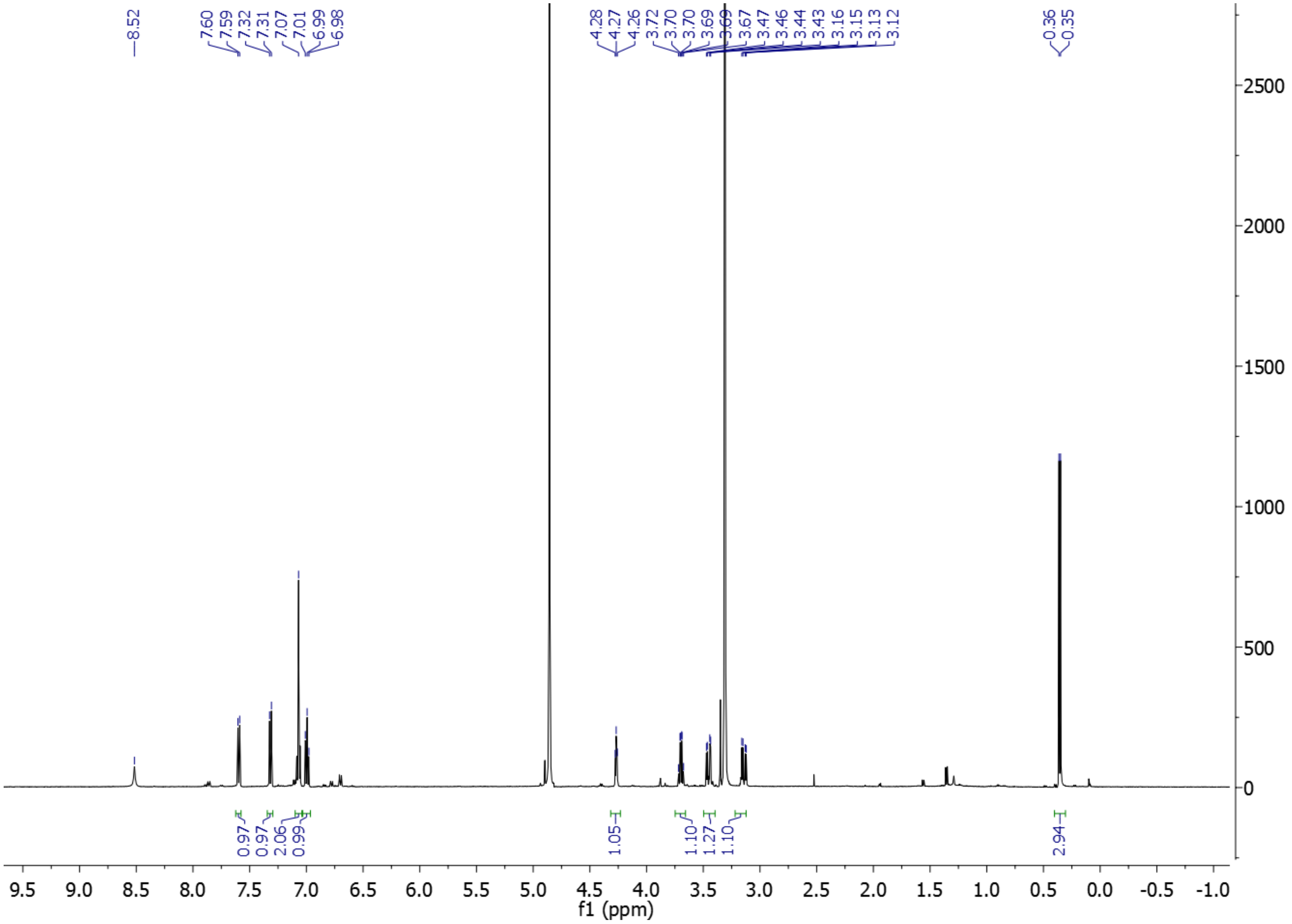

Figure S19. ^{1}H NMR spectrum of cyclo-L-Trp- L-Ala **(10)** in CD_3OD at 500 MHz.

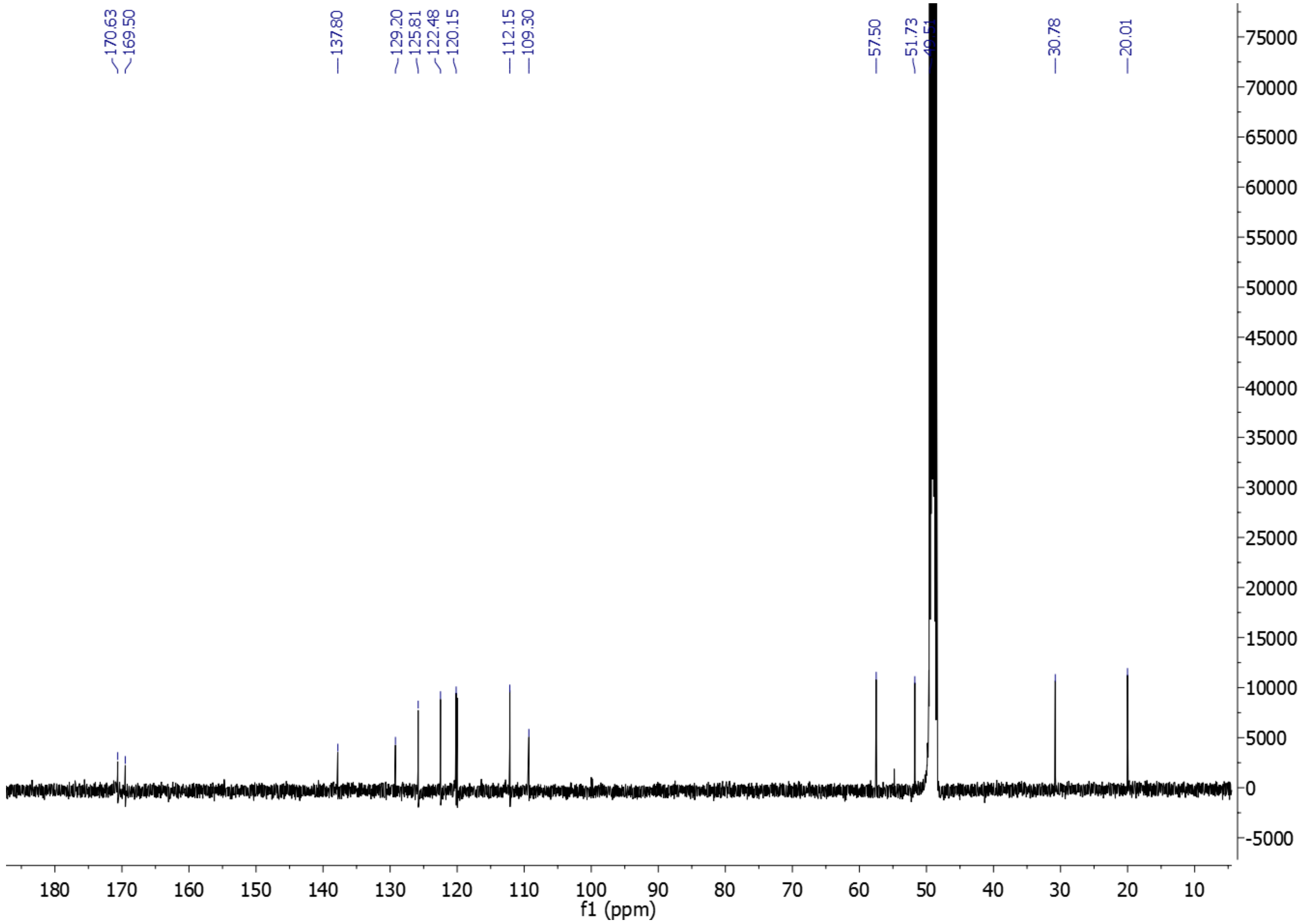

Figure S20. ^{13}C NMR spectrum of cyclo-L-Trp- L-Ala **(10)** in CD_3OD at 125 MHz.

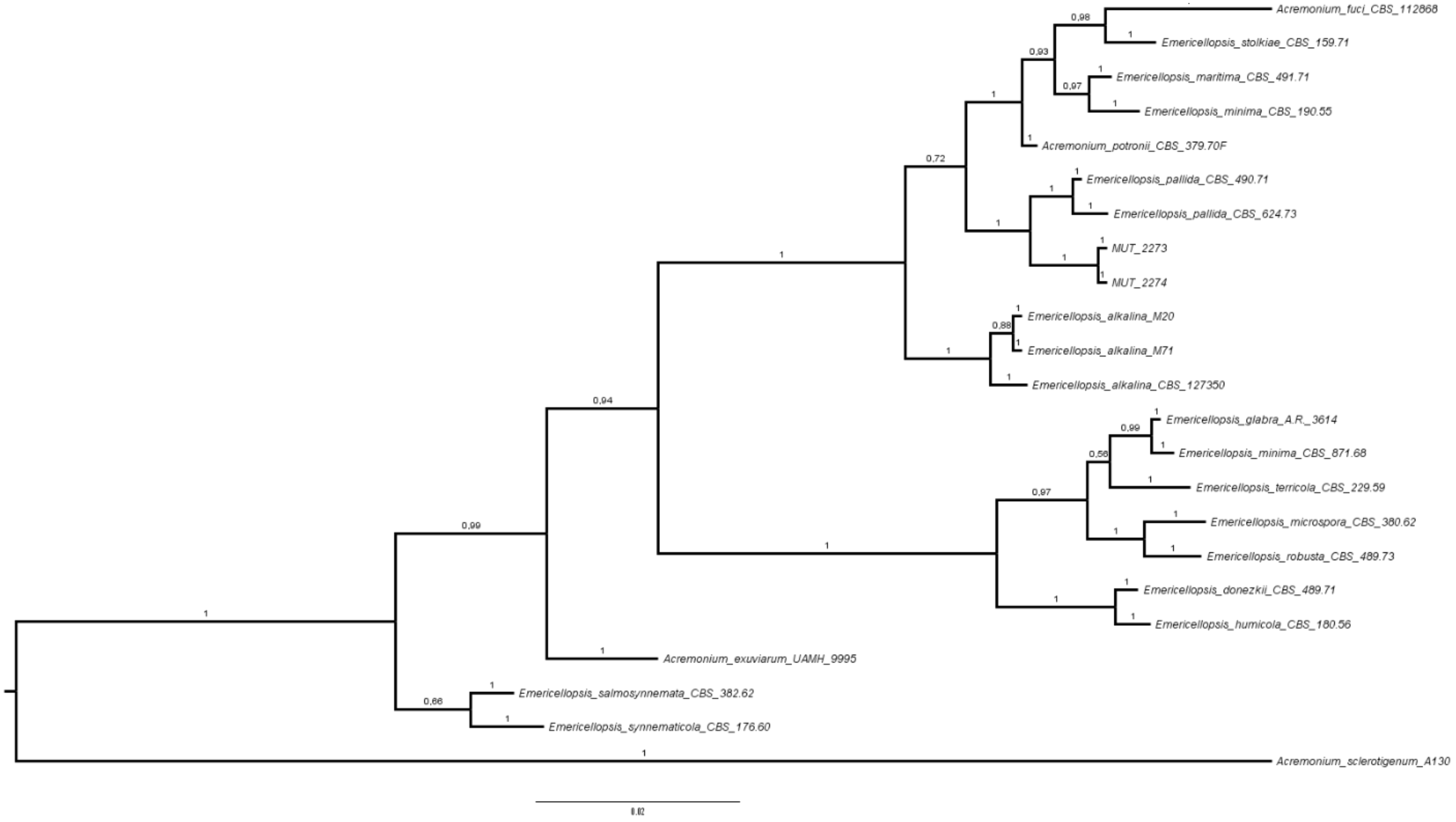

Figure S21. Bayesian phylogram of the genus *Emericellopsis* based on a combined dataset of ITS and beta tubulin partial sequences. MUT 2273 and MUT 2274 were identified as *Emericellopsis pallida*. Branch numbers indicate BPP values.

Table S4. Effect of different growth conditions on the HPLC-UV 280 nm chemical fingerprint of fungi, expressed as classes 1, 2, 3 and 4 at the increasing of the metabolic diversity. Exclusive peaks of each condition indicated in parenthesis. The changing in colours from dark to light blue and white indicated the increasing of the metabolic diversity.

MUT code	Taxa	Pure culture						Co-culture					
		WH	WHS	PDA	PDAS	GA	GAS	WH	WHS	PDA	PDAS	GA	GAS
2288	*Ceriporia lacerata*	2	1	1	1	1	1	1 (1)	1	1	1	1	1
2307	*Cladosporium allicinum*	2 (2)	2	2 (2)	2	2	1	1	1	2 (1)	2	2	1
2313	*Cladosporium allicinum*	2 (2)	2 (1)	2	2 (1)	2	2	2 (2)	2	1	1	2	2
2314	*Cladosporium cladosporioides*	3	2	2	2	1	1	2	1 (1)	2	2	1 (3)	1
2315	*Cladosporium pseudocladosporioides*	2 (1)	1	2 (1)	1 (2)	2	2	1	2	2 (1)	2	2 (2)	1
2282	*Coprinellus* sp.	2 (1)	1 (1)	1	1 (1)	1	1	1	1	1	1	1	1
2332	*Coprinellus* sp.	2	1 (1)	1	1	1	1	1	1 (1)	1 (1)	2	1	1
2273	*Emericellopsis pallida*	2	2	4 (1)	3	2	1	1 (2)	1 (1)	3 (3)	3	1	1
2274	*Emericellopsis pallida*	1 (1)	1 (1)	2	1	1	1	1	1 (1)	2	1	1 (2)	1
2316	*Eurotium chevalieri*	4	4 (1)	4 (1)	4 (4)	1	1	4	4 (2)	4 (3)	4	1 (1)	1(1)
2334	*Euthypella scoparia*	2 (2)	1 (1)	1	1	1	1 (1)	1 (3)	1	1	1 (1)	1	1
2317	*Fusarium solani*	1	1	4	2	2	1	1	1	3	2	2	1
2321	*Penicillium chrysogenum*	1	2	2	4	1 (1)	1	3	2	4	3	1	1
2328	*Penicillium oxalicum*	4	3 (1)	3	3	2	2	3 (1)	4 (2)	4	3 (1)	1	1
2322	*Penicillium paneum*	2 (1)	2	2	3	1	1	2	2	3	3	2	1
2326	*Penicillium paneum*	2	2	2 (2)	2	1	1	2	2	2	2	1	1
2331	*Psathyrella candolleana*	2 (2)	1	1	1	1	1	2	2	3	1	1 (1)	1
2415	*Rhodotorula mucillaginosa*	2	2	1 (1)	1 (1)	1	1	1	2	1	1	1	1
2413	*Tolypocladium cylindrosporum*	1 (1)	2 (1)	2	2	2	2	2	2 (1)	2	2 (1)	1	1
2410	*Tolypocladium cylindrosporum*	3 (1)	2 (2)	2 (2)	1 (1)	2	2	3	2	1	2	2 (1)	1

Table S5. Effect of different growth conditions on the development of the fungus (-, +, ++, +++); production of exudates ***(e)***, soluble pigments ***(s)*** and high sporulation ***(h)***; fungal-bacteria interaction as antagonistic response ***(a)***, predominance of the fungus ***(p)*** or complete inhibition of the bacterial growth ***(n)***. The changing in colours from dark to light blue and white, underlined the increasing of growth classes.

MUT code	Taxa	Pure culture						Co-culture					
		WH	WHS	PDA	PDAS	GA	GAS	WH	WHS	PDA	PDAS	GA	GAS
2288	*Ceriporia lacerata*	+++ *(e, s)*	++	+++	+++	+++	+++	+++ *(e, s, p)*	+++	+++ *(p)*	+++ *(n)*	+++	++
2307	*Cladosporium allicinum*	+++ *(h)*	+++ *(h)*	+++ *(h)*	+++ *(h)*	++ *(h)*	++	++ *(h, a)*	++ *(h, a)*	++ *(h, a)*	+++ *(h, p)*	+ *(h)*	+
2313	*Cladosporium allicinum*	+++ *(h)*	+++ *(h)*	+++ *(h)*	+++ *(h)*	++ *(h)*	+	+++ *(h, a)*	+++ *(h, a)*	+++ *(h, a)*	+++ *(h, a)*	++ *(h)*	+
2314	*Cladosporium cladosporioides*	+++ *(h)*	+++ *(h)*	+++ *(h)*	+++ *(h)*	+++ *(h)*	++	+++ *(h, a)*	+++ *(h, a)*	+++ *(h, a)*	+++ *(h, p)*	++ *(h)*	++
2315	*Cladosporium pseudocladosporioides*	+++ *(h)*	+++ *(h)*	+++ *(h)*	+++ *(h)*	++ *(h)*	+	+++ *(h, a)*	+++ *(h, a)*	+++ *(h)*	+++ *(h, n)*	+ *(h)*	+
2282	*Coprinellus* sp.	+++	+++	+++	+++	+++	+	+++ *(p)*	++	+++ *(p)*	++	++	+
2332	*Coprinellus* sp.	+++	++	+++	+++	+++	-	++ *(p)*	+	+++ *(p)*	++	+	+
2273	*Emericellopsis pallida*	+++	+++	+++	+++	++	+++	+++ *(a)*	+++	+++ *(a)*	+++	++	++
2274	*Emericellopsis pallida*	+++	+++	+++	+++	+++	+++	+++ *(a)*	+++ *(p)*	+++ *(a)*	+++ *(p)*	++	++
2316	*Eurotium chevalieri*	+++ *(e, h, s)*	+++ *(h, s)*	+++ *(h, s)*	+++ *(h, s)*	+	++	++ *(e, h, s)*	+++ *(h, s, a)*	++ *(h, s)*	+++ *(h, s, n)*	+	++
2334	*Eutypella scoparia*	+++	+++	+++	+++	++	++	+++	+++	+++	++	++	-
2317	*Fusarium solani*	+++ *(s)*	+++	+++ *(s)*	+++	+++	+++	+++*(s)*	+++ *(p)*	+++*(s, p)*	+++ *(p)*	+++	+++
2321	*Penicillium chrysogenum*	+++ *(h, s)*	+++ *(h, s)*	+++ *(h)*	+++ *(h)*	+++ *(h, s)*	+++ *(h)*	+++ *(h, s, a)*	+++ *(h, s, a)*	+++ *(h)*	+++ *(h, n)*	++ *(h, s)*	++ *(h)*
2328	*Penicillium oxalicum*	+++ *(h)*	+++ *(h)*	+++ *(h)*	+++ *(h)*	++	++	+++ *(h, p)*	+++ *(h, p)*	+++ *(h, p)*	+++ *(h, p)*	+++	+++
2322	*Penicillium paneum*	+++	+++	+++ *(h)*	+++ *(h)*	++	+	+++ *(a)*	+++ *(a)*	+++ *(h, p)*	+++ *(h, p)*	+++	+
2326	*Penicillium paneum*	+++	+++	+++ *(h)*	+++ *(h)*	+++	++	+++ *(a)*	+++ *(a)*	+++ *(h)*	+++ *(h, n)*	+++	+
2331	*Psathyrella candolleana*	+++	+	+++	-	+++	-	++ *(a)*	+	+++ *(a)*	-	++	-
2415	*Rhodotorula mucillaginosa*	+++	+++	+++	+++	+	+	+++	+++	+++	+++	-	+
2413	*Tolypocladium cylindrosporum*	+++	+++	+++	+++	+++	+++	+++ *(a)*	+++ *(a)*	+++	+++	++	++
2410	*Tolypocladium cylindrosporum*	+++	+++	++	++	++	+	++	++	++	++	+	+
